# Identification and correction of time-series transcriptomic anomalies

**DOI:** 10.1093/nar/gkaf524

**Published:** 2025-06-30

**Authors:** Sophia A Campione, Christina M Kelliher, Cullen Roth, Chun Yi Cho, Anastasia Deckard, Francis Motta, Steven B Haase

**Affiliations:** Department of Biology, Duke University, Durham, NC. 27708 United States; Azenta Life Sciences, South Plainfield, NJ, 07080 United States; Department of Biology, University of Massachusetts Boston, Boston, MA 02125 United States; Department of Biology, Duke University, Durham, NC. 27708 United States; Department of Biology, Duke University, Durham, NC. 27708 United States; Geometric Data Analytics, Inc., Durham, NC, 27701United States; Department of Mathematical Sciences, Florida Atlantic University, Boca Raton, FL 33431 United States; Department of Biology, Duke University, Durham, NC. 27708 United States

## Abstract

Transcriptomic analyses performed in time series have uncovered many important insights into dynamic biological processes such as circadian rhythms, cellular developmental cycles, and the cell cycle. Some of these studies have revealed transcriptomic artifacts (STRIPEs), characterized by substantial changes in transcript levels across the transcriptome between a time point and its temporal neighbors. These changes are unlikely to reflect underlying biology as the magnitude of the change is too large to occur within the time interval and because every gene in the time point exhibits a substantial change. Furthermore, STRIPEs occur across species exhibiting different biology, do not occur in the same phase across replicate time-series experiments, and can vary between technical replicates of a single time point. Here, we demonstrate STRIPEs in five time-series transcriptomic datasets across different species, biological processes, and timescales. We describe a computational method to detect STRIPEs in time series using the Kolmogorov–Smirnov statistical test, allowing for unbiased, user-friendly detection of STRIPEs. Finally, we present three methods for STRIPE correction and demonstrate their efficacy. Using periodicity analysis to identify periodic genes, we find nearly 600 genes changed in periodicity labeling following successful STRIPE correction, indicating the large impact of STRIPE removal on downstream analysis.

## Graphical Abstract

**Graphical Abstract:**
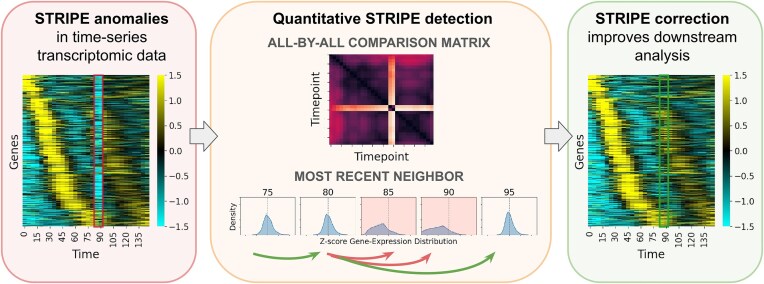

## Introduction

A major component of cellular state can be defined by the set of genes expressed and not expressed at a particular moment in time. Across the transcriptome, many phenotypes are controlled transcriptionally. Our current understanding of these transcriptional programs was enabled by technologies that measure the transcript level of every (or almost every) gene in a genome [1–3]. Moreover, performing these transcriptomic experiments in time series has revealed the importance of dynamics in multiple systems. Cells are dynamic: responding to intra- and extracellular signaling pathways to produce substantial changes in the transcriptome. Early time-series microarray experiments revealed a large program of phase-specific transcription during the budding yeast cell cycle [4, 5], and further studies indicated that a gene regulatory network might function as a cell-cycle oscillator [6–10]. Similarly, the gene regulatory network regulating the circadian clock in mammals controls up to 75% of the mammalian genome [11, 12].

As technologies advanced, researchers moved from microarray to RNA-seq platforms as they allow for full sequencing of the whole transcriptome and improved specificality compared to microarray analysis [13–16]. However, as our group made this switch, we noticed that a small number of time points in time-series RNA-seq experiments exhibited anomalous nonbiological expression levels across the entire transcriptome. We have called these anomalous points TRanscriptomic Irregular Profile Expression changes (STRIPEs) as they visually appear as a stripe on a heatmap of a time-series experiment. In each STRIPE, all transcripts in the transcriptome display elevated or depressed levels (or both) compared to neighboring points. As these anomalies present large-scale changes across the entire transcriptome, the presence of a STRIPE can substantially impair downstream analyses. Although we were not able to precisely pin down the origin of STRIPEs, to combat this challenge we instead have learned how to quantitatively identify and correct them to facilitate further analyses.

STRIPEs are most concerning where they cannot be easily identified or corrected. While STRIPEs can be identified in granular time series using our quantitative tools, in experiments where only a couple of samples are compared (e.g. pre/post-treatment or mutant/wild-type), it would be impossible to determine whether observed changes are biological or represent a STRIPE. This would be further exacerbated for experiments where only a subset of the transcriptome is interrogated. Identification of the origin of STRIPEs would greatly improve the ability to identify STRIPEs in experiments with limited samples. However, as we have not yet been able to identify the origin of STRIPEs, STRIPE identification and correction depend on comparison to non-STRIPE samples. Here, we describe the attributes of STRIPEs and describe a quantitative method for identifying them using comparison to neighboring/replicate samples. Additionally, we detail three methods for STRIPE correction to aid in downstream analyses.

## Materials and methods

### Strains, cultures, and synchronization

The four datasets include Yeast Cell Cycle 1 [17], Yeast Cell Cycle 2, Human Cell Cycle, and Human–*Plasmodium* Developmental Cycle [18]. Datasets for supplemental analyses include microarray Yeast Cell Cycle 1 datasets [9] and a STRIPE containing dataset from Wang *et al.* [19]. Datasets are described in Supplementary Table S1. A *Saccharomyces cerevisiae* strain derived from BF264-15D MATa bar1 [20, 21] was used for both *S. cerevisiae* datasets. In each case, yeast cultures were grown in rich YEP medium (1% yeast extract, 2% peptone, 0.012% adenine, 0.006% uracil) containing 2% dextrose. For the Yeast Cell Cycle 1 dataset, cells were grown at 30°C. For the Yeast Cell Cycle 2 dataset, cells were grown at 38.5°C. For both yeast datasets, cultures were grown overnight, arrested using 35–50 ng/μl of alpha-factor mating pheromone for 1.5 and 2.5 h, respectively, and then released by resuspending into fresh medium at their respective temperature (30°C or 38.5°C, respectively). Time points were collected at intervals of every 5 min for 245 min for the Yeast Cell Cycle 1 dataset and every 10 min for 290 min for the Yeast Cell Cycle 2 dataset. For the Human Cell Cycle dataset, the K562 human cell line was used. The cells were grown in RPMI + 10% bovine calf serum + P/S. The cells were synchronized via centrifugal elutriation and resuspended to begin the time course. Time points were collected at intervals every 2 h for 48 h. The Human–*Plasmodium* Developmental Cycle dataset was collected as described in [18]. Time points were collected every 3 h for 45 h. For each time course, samples were collected for RNA sequencing at each time point as described below.

### RNA isolation and RNA sequencing analyses

In each case, RNA was extracted via an acid phenol protocol described previously [22]. The microarray datasets were collected as described in [9]. For each RNA sequencing dataset, total RNA was isolated at each time point and sequenced at Duke Sequencing and Genomic Technologies. In each case, raw fastq files obtained from the sequencing core were aligned to the respective genome using STAR [23] or Salmon [24]. Aligned reads were quantified and normalized using one of three quantification methods: Cufflinks2 [25], Salmon [24], and RSEM [26], as additionally described in Supplementary Table S1. The Yeast Cell Cycle 1 dataset was aligned and quantified using STAR + Cufflinks2 with samples normalized together using CuffNorm, STAR + RSEM, and Salmon for comparison. The Yeast Cell Cycle 2 dataset was aligned and quantified using STAR + RSEM. The Human Cell Cycle dataset was aligned, quantified, and normalized using STAR + Cufflinks2. The Human–*Plasmodium* Developmental Cycle dataset was aligned, quantified, and normalized using STAR + RSEM as described in [18]. In [18], an additional cleaning step (including quantile normalization) was performed; however, here the “raw” data from RSEM were used. The resulting output from the Cufflinks2 analysis was in fragments per kilobase of transcript per million mapped reads (FPKM), and the output from RSEM provided both FPKM and transcripts per million (TPM) transcript level data. In each case, a single dataframe is produced containing the transcriptional data for each gene at each time point.

### STRIPE detection and correction

The STRIPE detection function (contained in Supplementary File S1 and present on the GitLab: https://gitlab.com/haase-lab-group/stripe_anomalies) uses the SCIPY Python package [27] and the ks_2samp two-sample Kolmogorov–Smirnov (KS) distance function [27–30]. The input for the STRIPE detection function is a transcriptional time course data frame and a user-supplied KS distance or *P*-value threshold. The function first performs a KS test comparing the first and second time points. If the metric is below the threshold, then comparison proceeds in the forward direction. However, if the metric exceeds the threshold, then the comparison occurs in the reverse direction, to allow for comparison to a non-STRIPE. The detection function thus assumes that both the first and second time points are not STRIPEs. In either direction, the function sequentially compares each time point to the most recent non-STRIPE neighbor. If the previous five time points are all identified as STRIPEs, then the comparisons are repeated in the reverse direction. Following comparison, the user-supplied KS metric threshold is used to designate STRIPE identity (0 = non-STRIPE, 1 = STRIPE). To aid in threshold selection, we developed a function to plot the distribution of KS distance statistics. As STRIPE identity in the STRIPE detection function depends only on one KS test, to further identify STRIPEs, a function is provided to display KS metrics (*P*-values and distance statistics) for all pairwise comparisons.

STRIPE correction can be performed via interpolation, quantile normalization, or *Z*-score quantile normalization. For interpolation, STRIPE time points were dropped and then interpolated using the PCHIP (Piecewise Cubic Hermite Interpolating Polynomial) method supplied in the Python pandas package [31]. The interpolated time points for the Yeast Cell Cycle 1, Yeast Cell Cycle 2, and Human Cell Cycle datasets were [145, 175, 180], [110, 140, 150], and [16, 18], respectively.

Quantile normalization was performed by using a quantile normalization function on the pandas dataframe containing the raw data for each dataset. More details can be found in the Supplementary data. As many downstream analyses require *z*-score normalization, quantile normalization was performed both before any necessary *z*-score normalizations (for simplicity called quantile normalization) and after *z*-score normalization (called *z*-score quantile normalization).

### Analysis and visualizations

**Figure 1.**
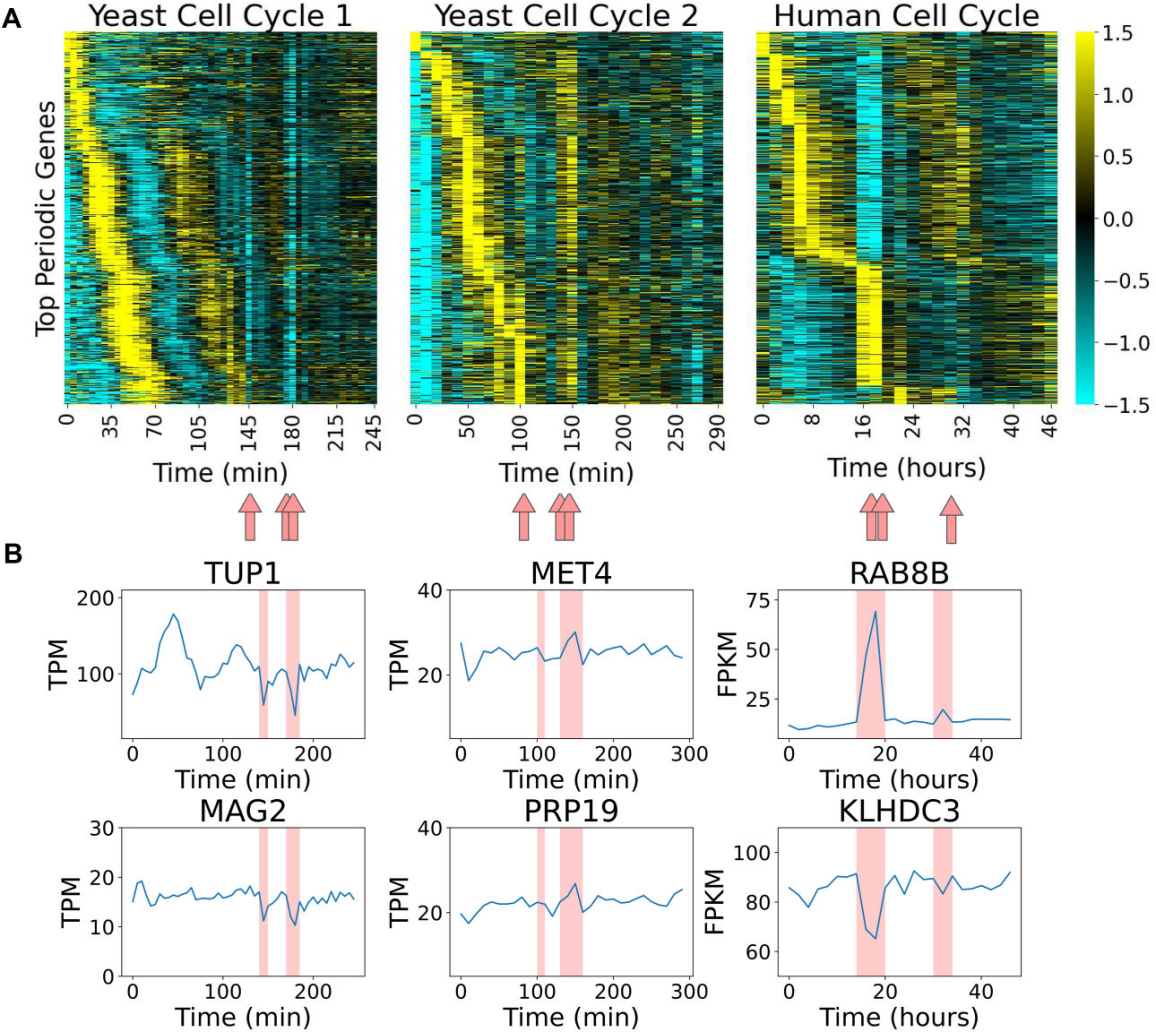
Visual identification of STRIPEs in time-series transcriptomic data. (**A**) Heatmaps depicting the *Z*-score normalized transcript profiles for the top 1000 periodic genes as ranked by JTK_CYCLE for each of the following datasets: Yeast Cell Cycle 1, Yeast Cell Cycle 2, and the Human Cell Cycle. The color bar corresponds to the *Z*-score: 1.5 (yellow) to −1.5 (blue). Red arrows indicate visually identified STRIPEs for each dataset: time points 145, 175, and 180 for Yeast Cell Cycle 1, time points 100, 140, and 150 for Yeast Cell Cycle 2, and time points 16, 18, and 32 for the Human Cell Cycle. (**B**) Line plots for transcript abundance of representative genes in each dataset are expressed in TPM or FPKM for each of the datasets. Visually identified STRIPEs are highlighted in red.

**Figure 2.**
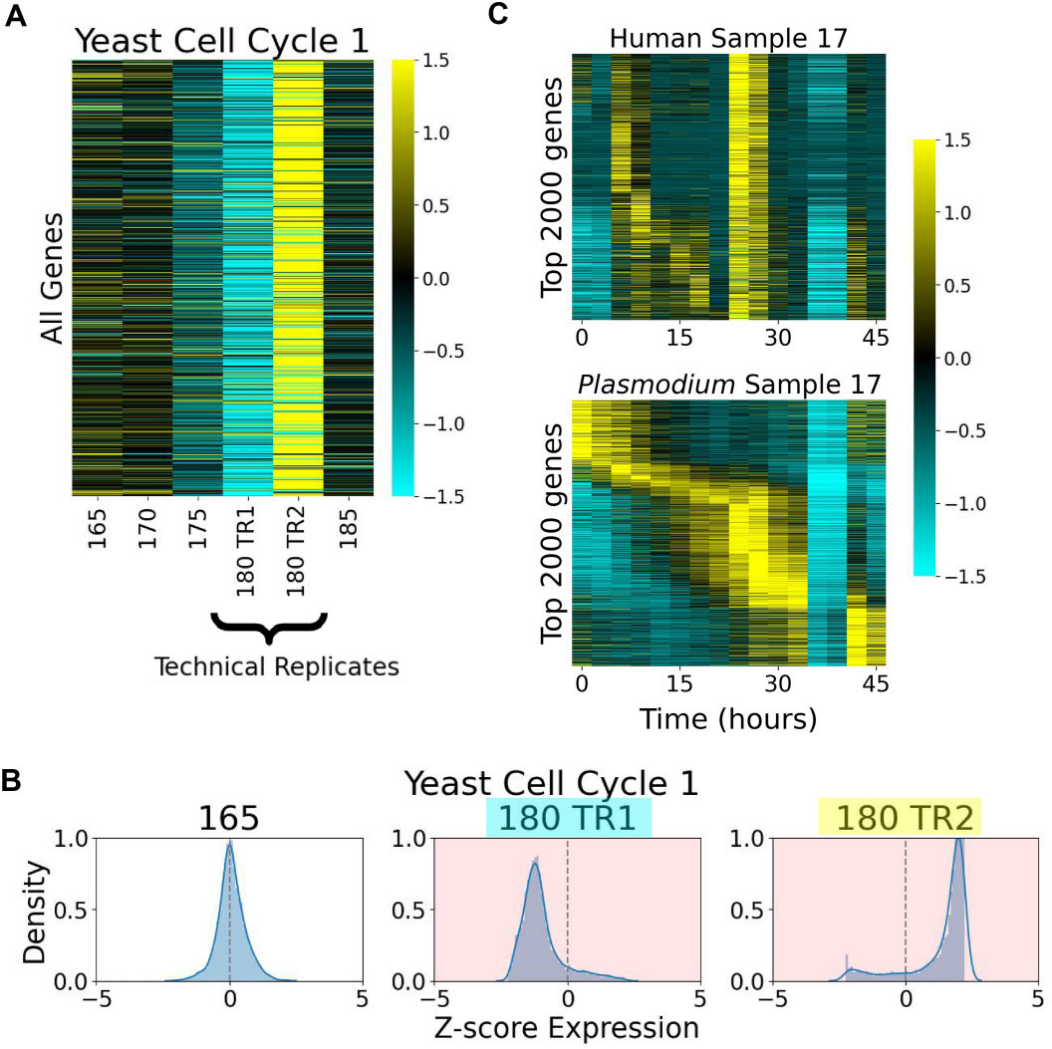
The origin of STRIPEs is technical. (**A**) Technical replicates at time point 180 (180 TR1 and 180 TR2) for the Yeast Cell Cycle 1 dataset both show dramatically different transcript dynamics across the entire transcriptome, as both exhibit a STRIPE. (**B**) Distributions of the expression *Z*-score for non-STRIPE time point 165 compared to both STRIPE technical replicates 180 TR1 and 180 TR2 in the Yeast Cell Cycle 1 dataset. (**C**) Top 2000 period genes human patient 17 infected with *Plasmodium vivax*. The transcriptomics for the human are in the top row and for the parasite in the bottom row.

Transcriptional line graphs for individual genes were generated using the raw (non-*Z*-score normalized) data. Transcriptomic heatmaps were generated using *Z*-score normalized data and plotted using the seaborn Python package [32]. For heatmaps showing dynamic behavior (Figs 1, 2C, and 4B), periodic genes were identified using JTK_CYCLE and then ordered on the first period max of expression. Transcript level distribution plots were plotted using the *Z*-score normalized transcript level data and the Python package seaborn [32]. KS distance distribution plots were generated using the distribution of KS distances for the comparisons applied by the STRIPE detection function. The KS distance matrix heatmap was generated using the KS distances for each pairwise comparison. Visualizations were performed using matplotlib (version 3.7.2) [33].

To determine the over-representation of genes aligned with the STRIPE, first the most periodic genes were identified using the JTK_CYCLE [34], DLxJTK [35], and Lomb–Scargle [36–38] periodicity algorithms. Next, genes were identified for which the transcriptional peak or trough was located at a STRIPE. The over-representation was calculated using the percent of periodic genes aligned with the STRIPE. *T*-tests were performed using a paired *t*-test in the R statistical programming environment (function t.test with argument: paired = T; R Core Development) [39]. STRIPE detection was performed using the STRIPE detection function and visual inspection. Python file containing analyses and visualizations is available on the GitLab: https://gitlab.com/haase-lab-group/stripe_anomalies.

## Results

### RNA sequencing artifacts appear in time-series transcriptomic datasets

Here, we present four distinct RNA-seq time-series datasets that presented with STRIPEs: (i) Yeast Cell Cycle 1 (optimized conditions) [17], (ii) Yeast Cell Cycle 2 (mild heat stress), (iii) Human Cell Cycle (Cho and Haase, unpublished), and (iv) Human–*Plasmodium* Developmental Cycle [18]. These datasets include dynamics of different timescales across different periodic processes in different species, documenting that STRIPEs can appear in biologically diverse datasets. The detailed protocols for each experiment are described in Supplementary Table S1. For each time-series experiment, at least eight time points per cycle were sampled. For each time point in each dataset, RNA was extracted, sequenced, and aligned as described in Supplementary Table S1.

To examine how STRIPEs impact the analysis of periodic dynamics, we focused on the set of periodically expressed genes. The periodicity detecting algorithm JTK_CYCLE [34] was used to identify the top 1000 periodic genes for each dataset. Periodic genes were ordered by peak time and plotted in a heatmap. The data are presented as *Z*-scores, with transcript levels at, above, and below the gene’s mean transcript level represented by black, cyan, and yellow, respectively (Fig. 1A). Visually, STRIPEs present as vertical stripes with substantial deviations from the expected transcriptional dynamics. In each dataset, at least one STRIPE can be visually identified, with three distinct types of STRIPEs: low, high, and bimodal in the Yeast Cell Cycle 1, Yeast Cell Cycle 2, and Human Cell Cycle datasets, respectively (Fig. 1A). These phenomena are observed in datasets collected across labs and over several years (Figs 1 and 2, and Supplementary Fig. S1) [17–19, 40]. Although we show only the most periodic genes, the STRIPE is present across the entire transcriptome (Supplementary Fig. S2). These STRIPEs can also be observed in line plots of individual genes (Fig. 1B). In these line plots, the STRIPEs appear as spikes in the expression curves (Fig. 1B).

For the Yeast Cell Cycle 1 dataset, time points 145, 175, and 180 display transcript levels that are lower than expected across the entire gene set as compared to neighboring time points. Visually, these low stripes correspond to a blue STRIPE that represents expression below the mean in the mean-normalized data (Fig. 1A) and sudden troughs in the line plots of representative genes (Fig. 1B). In the Yeast Cell Cycle 2 dataset, time points 140 and 150 appear to have elevated levels (high STRIPEs) across the transcriptome compared to their neighbors as seen by a yellow elevated expression STRIPE. Additionally, to a lesser degree, time point 110 shows a mild elevated STRIPE (Fig. 1A). These changes correspond to sudden peaks in the line plots of representative genes (Fig. 1B). In the Human Cell Cycle dataset, time points 16 and 18 show transcript levels that are either lowered or elevated for each gene compared to neighboring time points, resulting in a brighter blue and yellow bimodal STRIPE. To a lesser degree, time point 32 also shows a mild STRIPE (Fig. 1A). As the STRIPEs in the Human Cell Cycle dataset are bimodal, in line graphs they appear as either sudden troughs or peaks for representative genes (Fig. 1B).

### The origin of STRIPEs within RNA sequencing is technical rather than biological

Several observations suggest that STRIPEs have a technical rather than biological origin. The STRIPEs presented here (Figs 1 and 2, and Supplementary Table S1) occur during different periodic processes, at different timescales, and across different species, suggesting an origin unrelated to these biological functions. The timescale of the changes observed in Fig. 1 further suggests that these STRIPE time points are likely technical rather than biological, as transcript levels change substantially and at the next time point return rapidly to produce “spikes” in the data that are too rapid to be accounted for by transcriptional changes. In addition, STRIPEs are observed at time points across the entire transcriptome, ruling out the possibility that the phenomenon is related to noisy expression of a single gene.

To directly test whether STRIPEs were biological in origin, we compared two technical replicates of time point 180 from the Yeast Cell Cycle 1 dataset (Fig. 2A and B). The two technical replicates show dramatically different transcript levels across the entire transcriptome (Fig. 2A and Supplementary Fig. S3) and substantial changes in distribution of transcript level *Z*-scores across the gene set (Fig. 2B). Interestingly, though both technical replicates exhibit a STRIPE, they are of different subtypes: 180 TR1 is a low STRIPE, whereas 180 TR2 appears as a high STRIPE (Fig. 2A). The diverse expression values observed for the STRIPE at 180 suggest that the origin of the STRIPE is not biological but may have something to do with extraction or processing of that sample. Moreover, the STRIPE at time point 145 min occurring during the second cell cycle was not present in equivalent time points for experimental replicates of the time series, indicating that it likely is not a “normal” part of the dynamics observed in the time series (Supplementary Fig. S4).

We also examined Sample 17 from the Human–*Plasmodium* Developmental Cycle dataset, which consisted of *ex vivo* cultures of human blood infected with *P. vivax* [18]. This dataset contained RNA from two distinct species, sequenced together and then aligned to the respective genomes. Sample 17 exhibited STRIPEs at time points 36 and 39 in both species (Fig. 2C). Interestingly, the STRIPEs present at time points 24 and 27 were only visually identifiable in the human data. However, using the quantitative metric described below, at a certain threshold these were detected as STRIPEs for both species. However, these STRIPEs were not seen in any other of the participants’ samples (data not shown), indicating that these large-scale transcript level changes are not biological. Taken together, the data in Fig. 2 support a technical rather than biological origin underlying STRIPEs.

### The technical origin of STRIPEs

Though the origin of STRIPEs remains unknown, the possible areas of origin include RNA extraction, library prep and sequencing, and alignment and quantification. To determine the technical origin of STRIPEs, we looked for an association between metrics that measure the accuracy of the processes and STRIPEs. During RNA extraction, potential anomalies could originate from degraded RNA. To determine RNA quality, the RNA integrity number (RIN) score—an algorithmic measure of RNA integrity from several features of electrophoresis/electropherogram profiles—was used. For the Yeast Cell Cycle 1 dataset, STRIPEs had a lower RIN score compared to non-STRIPEs (*t*-test *P*-value = .0011). However, for the Yeast Cell Cycle 2 dataset, the opposite was found: STRIPEs had a higher RIN score compared to non-STRIPEs (*t*-test *P*-value = .0794). When samples from both datasets were taken together, STRIPEs had a moderately lower RIN score compared to non-STRIPEs (*t*-test *P*-value = .1033). However, this is likely because the Yeast Cell Cycle 1 dataset contained more time points than the Yeast Cell Cycle 2 dataset and thus made up the majority of the samples in this test. No correlation was found between concentration, 260/280 ratio, or the 260/230 ratio (*t*-test *P*-values of .6184, .9911, and .8402, respectively, for the combined Yeast Cell Cycle 1 and Yeast Cell Cycle 2 datasets). In the tests applied here, a clear correlation was not found (all *t*-test values found in Supplementary Table S2; more information found in Supplementary data).

Next, to determine whether the anomalies occurred during RNA sequencing, we evaluated RNA sequencing metrics (Supplementary Table S2). However, the RNA sequencing passed filter yield in base pairs, number of passed filter reads, Phred quality score (Q30%), and average quality score did not correlate with STRIPE identity (*t*-test *P*-values of .7007, .2644, .9695, and .9589, respectively). We also investigated whether the barcode sequence used for library prep for RNA sequencing could correlate with STRIPE identity. The barcode 5′-ACTGA-3′ mapped frequently to the S288C genome and was also found in two of the STRIPE samples in the Yeast Cell Cycle 1 dataset. However, the frequency of barcode mapping to the yeast genome did not correlate with STRIPE identity (Supplementary Table S3).

Finally, RNA alignment metrics were not consistently different between STRIPE and non-STRIPE samples. For the Yeast Cell Cycle 1 dataset, total reads, total reads aligned uniquely to the yeast genome, and total reads mapped to annotated genes were not significantly different (*t*-test *P*-values of .306, .381, and .236, respectively). For the Yeast Cell Cycle 2 dataset, most metrics showed insignificant differences between STRIPE and non-STRIPE samples (Supplementary Table S2). However, for this dataset, the total reads, the percent of reads that mapped uniquely, and the RSEM alignable percent values were significantly different (*t*-test *P*-values of .0194, .0232, and .0388, respectively), indicating a potential role in RNA-seq alignment and quantification.

To further investigate the role of RNA-seq alignment and quantification, we compared STRIPEs in the Yeast Cell Cycle 1 dataset using three different RNA-seq alignment and quantification pipelines. STRIPE presence and severity were determined using both visual inspection and the STRIPE detection function discussed below. The presence and location of STRIPEs remained consistent; however, the severity and visual appearance of the STRIPEs changed between these RNA-seq tools, indicating that these tools do play a role. Using STAR and Cufflinks [23, 25] for alignment and quantification produced minor bimodal STRIPEs at 145, 175, and 180 (Supplementary Fig. S5), whereas use of STAR and RSEM [23, 26] produced more pronounced lowered expression STRIPEs at these time points (Supplementary Fig. S5). Finally, use of Salmon for alignment and quantification showed increasing severity of the STRIPEs and additional STRIPEs at 170 and 190 also appeared (Supplementary Fig. S5). These changes indicate that alignment and quantification can play a role in the visual appearance of the STRIPE. Though the type of STRIPE was altered, the presence of a STRIPE remained consistent across these methods. These observations suggest that some unknown feature of RNA sequencing data prior to alignment and quantification could be introducing these errors. Alignment and quantification could then handle these errors in different ways to produce different visual output.

### Quantitative detection of STRIPEs in time-series transcriptomic data

Our findings suggest that STRIPEs likely have a technical rather than a biological origin. As these STRIPEs do not represent biological gene expression changes, detection and correction of STRIPEs enable a more accurate downstream analysis of the data. First, we sought to develop methods for quantitative detection of STRIPEs. While STRIPEs are identifiable by visual inspection, unwanted bias could be introduced into the analysis if STRIPE detection is left to subjective visual identification by individual investigators.

**Figure 3.**
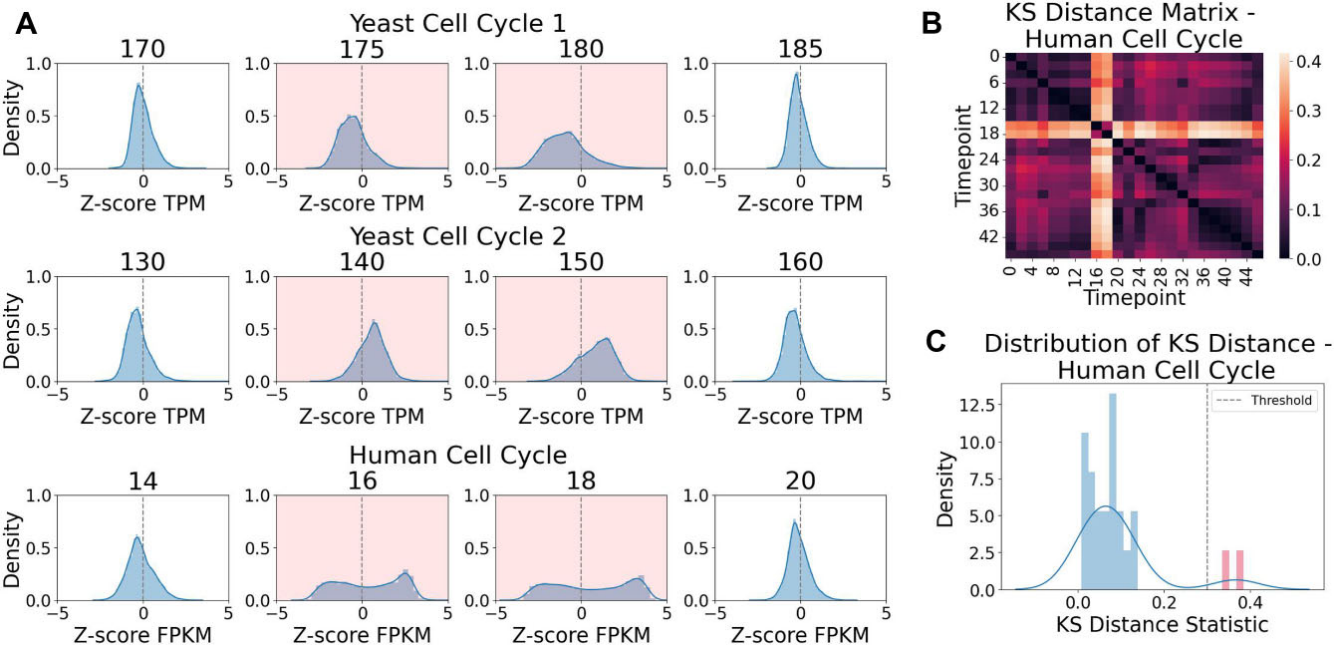
Quantitative Identification of STRIPEs. (**A**) *Z*-score transcript level distributions for representative STRIPEs and their non-STRIPE neighbors for the Yeast Cell Cycle 1, Yeast Cell Cycle 2, and Human Cell Cycle datasets. STRIPE time points distinguished by a pink background and flanking non-STRIPE time points are shown. (**B**) Pairwise KS distance comparisons between every time point in the Human Cell Cycle dataset are shown in a distance matrix. Color bar indicates distance with light colors having the greatest distance. (**C**) The distribution of KS distance statistics for the Human Cell Cycle dataset. For each time point the KS distance was calculated compared to the most recent non-STRIPE neighbor. Two outliers are highlighted in red. These outliers correspond to the visually identified STRIPEs at time points 16 and 18.

To alleviate this challenge, we have developed methods for quantitative STRIPE detection. STRIPEs are defined as nonbiological deviations in these distributions compared to temporally proximate samples (Fig. 3A and Supplementary Fig. S6). STRIPEs can therefore be identified by quantifying the KS distance between neighboring time points or technical replicates and selecting a threshold for this distance statistic. Interestingly, STRIPEs were also identifiable by distribution shape. Non-STRIPEs primarily showed a *Z*-score transcript level distribution with a peak centered around 0 (Fig. 3A and Supplementary Fig. S6), whereas STRIPEs exhibited a deviation from this trend (Fig. 3A and Supplementary Fig. S6).

Samples in an experiment can be compared in an “all-by-all” approach, where each set of samples is pairwise compared using a KS test [27–30]. The resulting KS distance statistics are plotted in a distance matrix. For the Human Cell Cycle dataset, STRIPEs were easily apparent using this method (Fig. 3B). However, in some cases non-STRIPE time points also deviate from the expected distribution shape. For example, time points taken during the recovery from synchronization in the Yeast Cell Cycle datasets also exhibit a leftward lean in the transcript level distributions (Supplementary Fig. S6A and B). Therefore, this all-by-all comparison matrix performed more poorly in STRIPE identification for these datasets (Supplementary Fig. S7).

Instead of an all-by-all comparison, STRIPE detection can be performed by comparing values to neighboring time points, as temporally proximate neighbors should show minor changes in distribution. The STRIPE detection function (STRIPE detector) quantifies changes in distributions by comparing each time point to their most recent non-STRIPE neighbor using a two-sample KS test to determine similarity. To check whether the first time point is a STRIPE, the detection function compares the first and second time points. If they show a significant difference, then the comparison occurs in the reverse direction (from the end of the time series to the front). However, if the last time point is also a STRIPE, the STRIPE detector will fail to establish STRIPE identity. In this case, individual time points should be dropped, or an all-by-all distance matrix should be performed instead. As the STRIPE detection function depends on comparison to the most recent non-STRIPE neighbor, multiple consecutive STRIPEs increase the temporal distance between compared samples. This could also increase the distance between distributions of these samples. In the case of five consecutive STRIPEs, the detection function will check the comparison in the reverse direction. However, if both directions show five consecutive STRIPEs, then the STRIPE detector may struggle to establish STRIPE identity and an all-by-all comparison should be made as shown in Fig. 3B.

At each comparison, the STRIPE detection function labels the time point as a STRIPE or non-STRIPE based on a user-input *P*-value or distance statistic threshold. Selection of this threshold can still be difficult, as all samples will differ from one another to at least a small degree due to biological noise. One method for threshold selection is plotting the distribution of the KS distance statistics and identifying outliers. For example, for the Human Cell Cycle dataset, time points 16 and 18 showed a substantially higher KS distance statistic compared to the rest of the time points in the distribution (Fig. 3C). Therefore, a threshold was selected to include these two points as STRIPEs. These two time points were also previously visually identified as STRIPEs (Figs 1 and 3A). For each dataset, the STRIPEs identified by visual inspection matched closely with those identified by the STRIPE detector function (Supplementary Fig. S7). However, when outliers are not apparent from the distribution plot, threshold determination can be difficult. Selection of a threshold in this case depends on the experimental designs and goals. To aid researchers in considering the stringency required, *P*-values can additionally be generated by the STRIPE detection function for review.

Furthermore, several factors should be considered when identifying STRIPEs in a dataset. Here, STRIPEs presented not only as a deviation from their neighbors, but also as a deviation from the expected zero-centered *Z*-score transcript level distribution. Although this deviation could be an indicator of a STRIPE, biological factors could conceivably alter the distribution. Therefore, both the all-by-all matrix comparison and the STRIPE detection function instead depend on comparison to samples for which large-scale changes in the transcriptome are unexpected biologically. However, in cases where threshold selection is difficult, comparisons could additionally be made to a zero-centered *Z*-score transcript level distribution. However, for most comparisons, we recommend comparing to replicate samples or temporally proximate samples, as there are biological factors that cause these distributions to deviate from a perfect zero-centered *Z*-score distribution.

These tools were originally designed to handle time-series transcriptomic data but can easily be used to compare between replicate samples. However, STRIPE detection cannot be accurately performed with an insufficient number of samples. Comparison between two samples, such as in experiments comparing pre- and post-treatment samples, is insufficient to determine whether the distance between samples is unexpected and which sample is the non-STRIPE sample. Even for experiments with three samples, it can be difficult to determine STRIPE identity. For example, if a single outlying sample is identified among three technical replicates, it is likely the STRIPE sample; however, it is possible that the two similar samples are both STRIPEs. With only three samples, it remains impossible to confidently determine which samples are STRIPEs. Each additional technical replicate and each additional time point improves power for STRIPE identification. The STRIPE detection method only identifies differences between samples. As these differences could also reflect true biological dynamics, STRIPE identification also relies on experimental design and interpretation that could reveal whether changes can be biological. For example, gene expression changes can only occur at a certain timescale, so samples can be collected more frequently to show that gene expression changes are not biological. Methods for STRIPE detection are included as Python functions in the Python utilities file (Supplementary File S1) and have been added in an update to the Inherent Dynamics Visualizer [41].

### Methods for correction of technical anomalies

With methods that could quantitatively identify STRIPEs, we aimed to develop methods for “correcting” the STRIPE points to restore the smooth curves expected for the time-series data. However, though we use the term “correction,” in many cases following STRIPE correction methods, the presence of the STRIPE was not fully removed (as discussed further below). Instead, STRIPE correction seeks merely to minimize the impact of these anomalies by returning the expression to an expression pattern as close as possible to the true biology. We show that this “correction” dramatically improves downstream analysis. Therefore, correction of STRIPEs is essential for effective downstream computational analyses and interpretations of the data. We considered two classes of corrections for STRIPE points, including interpolation and normalization approaches.

PCHIP interpolation is a method used to construct unknown new data points from a set of known data points [42]. This is often used for resampling or for reconstruction of missing data [43, 44]. For STRIPE correction, STRIPE time points can be dropped and reconstructed using PCHIP interpolation. This method does not utilize any information contained in the STRIPE time points. Therefore, this method makes two key assumptions: (i) STRIPE samples are sufficiently “flawed” such that they are not informative and (ii) the remaining time points in the experiment can inform the missing data. In this method, any informative transcriptional changes contained within the STRIPE samples are lost.

Another method for correcting STRIPEs is quantile normalization, a method commonly used in microarray studies. Quantile normalization corrects for systematic bias and technical variation by transforming distributions of each sample to have the same statistical properties [45–48]. In contrast to PCHIP interpolation, the quantile normalization method does utilize the data contained in STRIPE time points. Furthermore, it is performed across the entire dataset, rather than performed only on STRIPE time points. The key assumptions of this method are that (i) any variation originates from technical variation rather than from biological factors and therefore (ii) each sample should have the same statistical distributions [46]. When these assumptions are not met, features can be artificially introduced and biological changes can be obscured [45].

We chose quantile normalization because the assumptions appear to align with our findings regarding the distribution changes in STRIPE points (Fig. 3) and the technical origin of the STRIPE. Non-STRIPE time points are similar in distribution across the time series, in particular when compared to neighboring time points (Supplementary Fig. S6), whereas STRIPE time points show substantial deviations in these statistical distributions (Fig. 3A and Supplementary Fig. S6). The bulk of evidence indicates that STRIPEs are likely technical rather than biological in origin (Fig. 2). However, in cases where biological factors are expected to alter these distributions in informative ways, the assumptions made by quantile normalization would not be valid.

**Figure 4.**
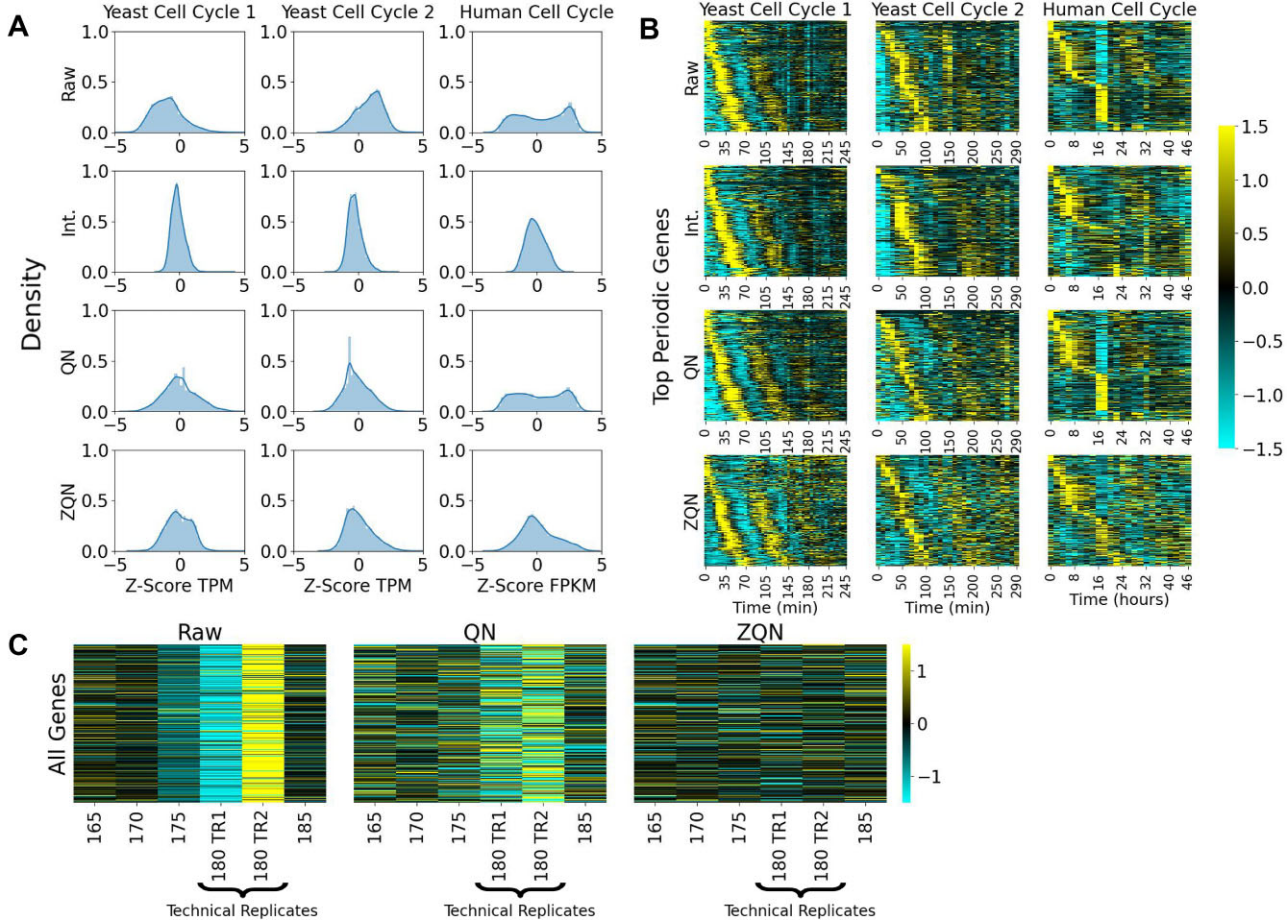
STRIPE correction methods reduce STRIPE appearance. (**A**) The *Z*-score transcript level distribution for a representative STRIPE time point is shown before STRIPE correction and following three STRIPE correction methods for the Yeast Cell Cycle 1, the Yeast Cell Cycle 2, and the Human Cell Cycle datasets. The three STRIPE correction methods include PCHIP interpolation (Int.), quantile normalization (QN), and *Z*-score quantile normalization (ZQN). (**B**) The *Z*-score normalized time-series heatmaps are shown before STRIPE correction (Raw) and following the same three STRIPE correction methods in panel (A). (**C**) The entire transcriptome for the Yeast Cell Cycle 1 dataset, including two technical replicates at TP 180 compared to flanking points before (Raw) and after (QN, ZQN) STRIPE correction using quantile normalization and *Z*-score quantile normalization.

STRIPE correction was evaluated using both visual inspection and quantitation by the STRIPE detector function. By visual inspection, the correction was considered successful for STRIPE time points that showed a reduction in appearance in the heatmaps and distribution plots following STRIPE correction (Fig. 4). Additionally, the correction was considered successful for STRIPE time points that no longer were detected at the same threshold by the STRIPE detector following STRIPE correction (Table 1).

**Table 1.** STRIPE identification

Dataset	Raw	Raw	Int.	QN	ZQN
	Visual inspection	Detector function	Detector function	Detector function	Detector function
Yeast Cell Cycle 1	145	145	None	None	None
	175	175			
	180	180			
Yeast Cell Cycle 2	100	0	0	None	None
	140	10	10		
	150	20	20		
		100			
		140			
		150			
Human Cell Cycle	16	16	None	16	None
	18	18		18	
	32				

STRIPEs identified in the Yeast Cell Cycle 1, Yeast Cell Cycle 2, and Human Cell Cycle datasets both prior to STRIPE correction (Raw) and following three STRIPE correction methods. The three STRIPE correction methods include PCHIP interpolation (Int.), quantile normalization (QN), and *Z*-score quantile normalization (ZQN). For the Raw data, STRIPEs were identified using both visual inspection and the STRIPE detector function. Following successful STRIPE correction, the number of STRIPEs identified by the function was reduced. Note that the 0, 10, and 20 min time points from the Yeast Cell Cycle 2 dataset were not interpolated.

PCHIP interpolation performed well on all datasets, with all STRIPEs showing a visual and quantifiable reduction following this correction method. This was seen by a reduction in the leftward, rightward, and bimodality of the distribution (Fig. 4A and Supplementary Fig. S6), a reduction in the appearance of the STRIPEs (Fig. 4B), and a lack of identification by the detector function at the same threshold (Table 1 and Supplementary Fig. S8). Time points 0, 10, and 20 were still detected in the Yeast Cell Cycle 2 dataset as these time points were not interpolated. Interpolation was performed only on time points identified by both visual inspection and the STRIPE detector function, as we suspected that time points 0, 10, and 20 could have a biological origin due to the release from synchronization.

However, quantile normalization performed inconsistently across datasets. Both Yeast Cell Cycle datasets showed full correction of all STRIPEs following quantile normalization (Fig. 4A and B, Table 1, and Supplementary Fig. S6A and B). However, the STRIPEs at time points 16 and 18 in the Human Cell Cycle dataset were not corrected as the bimodality of the distribution remained (Fig. 4A and Supplementary Fig. S6C), the appearance of the STRIPEs was not reduced in the heatmap (Fig. 4B), and the STRIPEs at time points 16 and 18 were still detected (Table 1). Similarly, a KS all-by-all comparison matrix showed persistence of the STRIPEs following quantile normalization.

Quantile normalization is performed on the raw data, followed by subsequent *Z*-score normalization for visualization and for the STRIPE detection function. Therefore, the definition of STRIPE depends on *Z*-score normalized data. As quantile normalization was not successful in correcting STRIPEs in all of the datasets, we additionally tested *Z*-score quantile normalization, where the data were first *Z*-score normalized and then quantile normalized. Therefore, the difference between the quantile normalization and *Z*-score quantile normalization methods lies only in the order of operations, much like the difference between RPKM/FPKM and TPM [49]. *Z*-score quantile normalization (ZQN) successfully corrected the STRIPEs in all three datasets (Fig. 4A and B, and Table 1). Notably, the STRIPEs in the Human Cell Cycle dataset that persisted following quantile normalization were successfully corrected using *Z*-score quantile normalization by visual inspection (Fig. 4A and B, and Supplementary Fig. S6C) and by the STRIPE detector function (Table 1). Additionally, the KS distance all-by-all comparison matrices showed a reduction of the STRIPE (Supplementary Fig. S8).

The two-quantile normalization STRIPE correction methods were additionally applied to the technical replicates 180 TR1 and 180 TR2 from the Yeast Cell Cycle 1 dataset (Fig. 4C). After both quantile normalization and *Z*-score quantile normalization, the appearance of the STRIPEs was dramatically reduced for both technical replicates, though the transcript levels between the two technical replicates were not identical even following normalization (Fig. 4C).

As described earlier, the different STRIPE correction methods make different key assumptions. As shown in Fig. 4, these methods also perform differently on experimental data. Therefore, selection of the correct STRIPE correction method is vital to ensure accurate downstream analysis. Where possible, minimally processed data are often preferred to avoid introducing bias. For experiments with changes in distributions confined to STRIPE samples, quantile normalization is the preferred method as it does not necessitate dropping information. Furthermore, in the case of multiple consecutive STRIPEs, interpolation may not be sufficient as remaining data contain large temporal gaps, thus also necessitating use of quantile normalization for STRIPE correction. However, in cases where quantile normalization does not correct the STRIPE, use of *Z*-score quantile normalization instead is recommended. In the case where neither quantile normalization method is sufficient for STRIPE correction, interpolation can then be introduced to ensure successful STRIPE correction. Interpolation is also the preferred method for STRIPE correction where the datasets are expected to violate the assumptions made by quantile normalization. For example, quantile normalization will obscure cases where biological factors introduce informative changes in distribution shape.

Each of these methods has been implemented in an update to the Inherent Dynamics Visualizer to allow for user-friendly data visualization and data cleanup [41], to allow for user-friendly STRIPE correction. Additionally, they are included as Python functions in the Python utilities file (Supplementary File S1).

### Correction of technical anomalies improves downstream analysis

**Figure 5.**
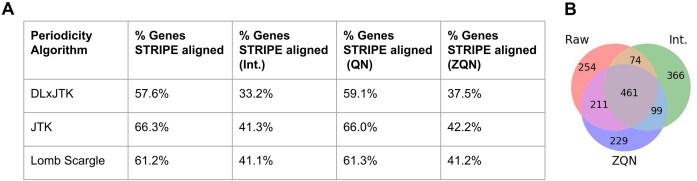
STRIPE correction improves periodicity analysis performance. (**A**) The top 1000 periodic genes were compared for the Human Cell Cycle dataset using DLxJTK, JTK_CYCLE, and Lomb–Scargle between the raw data and the three STRIPE correction methods. The percentage of genes for which a biological peak or a trough fell in line with the STRIPE time points 16 and 18 was quantified. (**B**) Venn diagram comparing the number of genes identified in the TOP 1000 by JTK using the raw data, the interpolated data (Int.), and the *Z*-score quantile normalized data (ZQN) for the Human Cell Cycle dataset.

Reproducible analyses of transcriptomic data rely on the assumption that samples are relatively free of noise, with any changes between samples stemming entirely from biological origins. A common analysis for the cell cycle and circadian fields includes identification of periodic genes as shown in Fig. 1. The presence of STRIPEs can confound periodicity analysis by over-representing genes for which the biological dynamics fall in line with the false peaks and troughs introduced by the STRIPE (Fig. 5). Conversely, genes for which the STRIPE interrupts the biological dynamics will be underrepresented, as the STRIPE would obscure the true periodic behavior of the genes. Therefore, transcriptome changes introduced by technical anomalies such as STRIPEs can impede downstream analysis.

To ask whether STRIPE detection and correction can alleviate issues with downstream analyses, we investigated the effect of STRIPEs on periodicity analysis using common periodicity tools JTK_CYCLE [34], DLxJTK [35], and Lomb–Scargle [36–38]. Using the Human Cell Cycle dataset, the top 1000 most periodic genes by each periodicity ranking algorithm were selected. Assuming peak time is uniformly distributed across the time points over a single cell cycle (in this case, 12 time points), we would expect ∼17% of the genes to fall in line with the two STRIPEs for the Human Cell Cycle dataset. While peak-time-based phase assignment remains inconsistent across studies [50], this assumption is not unreasonable. For human cell cycle, the percent of genes peaking in each phase was previously shown to be uniformly distributed: between 16.8% and 24.1% of cell-cycle genes were shown to peak in each phase [51]. In contrast, the percentage of genes in line with the STRIPEs at time points 16 and 18 represented around 62% of all genes (Fig. 5A). This indicates an over-representation of genes in line with the STRIPE time points. Following STRIPE correction, the over-representation was reduced from 62% to 39% and 40% for interpolation and *Z*-score quantile normalization, respectively (Fig. 5A), indicating an improvement of periodicity analysis following successful STRIPE correction. Notably, these percentages were not fully restored to the expected 17%. As also observed in Supplementary Fig. S8, STRIPE “correction” does not fully remove the impact of the STRIPEs but rather minimizes their impact to improve downstream analysis. Since quantile normalization performed poorly on the Human Cell Cycle dataset (Fig. 4), as expected, the over-representation was not improved for this method (Fig. 5A).

In another analysis, the top 1000 genes as ranked by JTK_CYCLE were compared using the Human Cell Cycle dataset between the raw, interpolated, and *Z*-score quantile normalized data. There was a large overlap of 461 genes between the three methods (Fig. 5B). However, there was also a large set of genes that were nonverlapping (Fig. 5B). The set of genes unique to a correction method further indicates that the presence of STRIPEs interferes with the ability of periodicity algorithms to accurately rank the periodicity of the genes. As STRIPEs lead to an over-representation of genes in a specific phase that fall in line with the STRIPE, periodicity analyses will overlook many important periodic genes not in line with the STRIPE. Moreover, downstream analyses that depend on proper identification of top periodic genes, such as network inference tools [41, 52], will also be obscured. Therefore, correction of STRIPEs is integral to accurate analysis of the data.

Many other potential downstream analyses could be obscured by the presence of a STRIPE. While this paper cannot explore all potential downstream analyses, we additionally used a Python implementation of DESeq2 [53, 54] to identify differentially expressed genes between STRIPE and non-STRIPE samples. Though there is no biological difference distinguishing STRIPE and non-STRIPE (as discussed previously), a significant number of genes are differentially expressed between the STRIPE and non-STRIPE samples (Supplementary Table S4). This indicates that STRIPE presence can substantially impede such analyses.

Another option for handling STRIPE anomalies is dropping the affected samples. For some experiments, information from neighboring time points or replicate samples could be sufficient for analysis. For example, in the Yeast Cell Cycle 1 dataset, the STRIPEs primarily obscure the third cycle, whereas periodicity can be verified with two cycles [55]. Therefore, sufficient information is contained in the Yeast Cell Cycle 1 dataset even when all STRIPEs are dropped. However, this is not always possible. Many experiments do not have sufficient samples. In which case, the loss of a sample could be detrimental to analysis. For example, many experiments would not have technical replicates or neighboring time points to utilize. In these cases, use of the transcriptional data within the STRIPE would be necessary for any analysis to be possible.

Even for time-series experiments, some analysis may depend on regularly sampled time series. For example, while Lomb–Scargle [36–38, 56] can handle irregularly sampled time series, this is not true of all analysis algorithms [55]. For example, DLxJTK used in Fig. 5 cannot be run on irregularly sampled data. This necessitates STRIPE correction rather than STRIPE removal. Interpolation drops the STRIPE time points and “reconstructs” them using information from neighboring time points, thus allowing for missing data to be filled in for analysis. However, as previously discussed, any informative transcriptional changes contained within the STRIPE samples are lost. STRIPE correction via quantile normalization allows for these potentially key insights to be kept, while correcting the confounding influence of the technical anomaly.

## Discussion

Time-series transcriptomic analyses provide temporal information regarding dynamic processes. These time-series transcriptomic studies have revealed STRIPEs: technical anomalies that correspond to erroneous large-scale transcriptomic differences between samples. We show these STRIPEs have a technical rather than biological origin. As these STRIPEs do not represent true biology, their identification and correction are integral to proper interpretation and downstream analysis. We have developed a quantitative method for STRIPE detection and three methods for STRIPE correction in time-series transcriptomic datasets to diminish these concerns. The methods for STRIPE correction include PCHIP interpolation, quantile normalization, and *Z*-score quantile normalization. Interestingly, we rarely observed stripes in time-series transcriptomic experiments using microarrays. This discrepancy could be related to a quantile normalization step frequently included in microarray processing pipelines to remove systematic bias and technical variation. While some RNA-seq pipelines and analyses do employ quantile normalization, it is a step that is frequently absent in RNA sequencing studies [57–59]. Consistently adding quantile normalization (specifically *Z*-score quantile normalization) to RNA-seq processing pipelines could prove a crucial step to reducing the presence of STRIPE artifacts in RNA-seq data. Additionally, other normalization procedures could also minimize the impact of STRIPEs on downstream analysis. Here, we focus on highlighting a potential problem, rather than a comprehensive analysis of additional normalization methods. *Z*-score quantile normalization successfully corrected the STRIPES; however, STRIPEs remained in the Human Cell Cycle dataset following quantile normalization alone (Fig. 4B). The normalization procedure in CuffNorm did not successfully correct STRIPEs for the Yeast Cell Cycle dataset processed using Cufflinks2 and CuffNorm (Supplementary Fig. S5). Similarly, the normalization implemented by DESeq2 did not correct STRIPE presence, as a substantial number of differentially expressed genes were still detected between STRIPE and non-STRIPE samples even following DESeq2 normalization (Supplementary Table S4). In contrast, successful STRIPE correction using interpolation reduces the differential expression between STRIPE and non-STRIPE samples substantially (Supplementary Table S4).

We have demonstrated that STRIPE correction enables more accurate analysis of the data, by minimizing the impact of STRIPEs on downstream analysis and visualization. To aid in user-friendly STRIPE detection and correction for more accurate analysis of transcriptomic data, we have incorporated the STRIPE detection and correction functions into a “Data Visualization and Cleanup” update to the Inherent Dynamics Visualizer [41]. STRIPE detection and correction depend on the use of either technical replicates or closely neighboring time points.

However, these methods also have limitations. First, as seen in Fig. 5 and Supplementary Fig. S8, STRIPE correction does not fully correct the impact of the STRIPE, but rather minimizes the impact. Once the technical origin is understood, additional efforts should be made to avoid STRIPEs or attempt full STRIPE correction. Additionally, because we do not yet understand the technical origin of STRIPEs, detection of STRIPEs depends on comparison to additional samples (either technical replicates or temporally proximate neighbors). Determining whether changes identified by the STRIPE detector are biological or technical in nature relies on experimental design and interpretation. STRIPEs are most concerning in experiments in sparse time series with a limited number of samples. Due to cost limitations, many transcriptomic studies are performed using few samples—with limited replicates and/or infrequent time points—which can introduce bias and impair reproducibility [60–66]. For example, studies may only sample twice: before and after treatment. With only two samples, STRIPEs would be indistinguishable from true biological dynamics, as any changes identified at longer timescales could be biological or nonbiological in nature. At shorter timescales, where large-scale gene expression changes would be biologically infeasible, we could determine whether a STRIPE was present; however, determining which sample was a STRIPE would be impossible without another point of reference.

Though the STRIPE detection function can identify changes even with very limited numbers of samples, determining the origin of these changes remains difficult or impossible with sparse sampling. Introduction of even just two technical replicates at each point improves the confidence of STRIPE detection. Previously it has been suggested that six technical replicates per condition is the “gold standard” for RNA-seq experimental design [63]. For periodic biological processes, though increasing the number of replicates does increase statistical power, increasing temporal resolution performs even better [34, 60, 67]. With only a few time points per cycle, it can be difficult to distinguish between biological transcriptome changes and those introduced by STRIPEs. Analysis is typically improved by use of at least 10–12 samples per cycle [22, 60]. To test the sensitivity of the detector to sample number, we performed a leave-one-out analysis where time points from the HCC time series were left out to calibrate STRIPE detector performance in time series with fewer time points. Though STRIPE detection was possible with more limited sample numbers and differences between time points were always detectable, in general, STRIPE detection is improved by increased sample number (Supplementary Fig. S9). Note that the detection of sample 16 was still detectable when only every fourth point was used (Supplementary Fig. S9C), and that the loss stripe detection of sample 18 was due to its omission when every other time point was dropped (Supplementary Fig. S9B). To prevent false identification of large transcriptome changes resulting from these technical anomalies, we highly encourage increased sampling frequency and/or incorporation of technical replicates.

STRIPEs likely originate from one (or several) of the steps in the collection and sequencing process to generate time-series transcriptomic data: RNA extraction, library construction, RNA sequencing, and/or alignment and quantification. From the common metrics collected in these three steps, we have not yet found a well-correlated indicator that would suggest a technical origin and thus a potential fix. Some STRIPEs were conserved between the two species, indicating a likely technical origin (Fig. 2C). That said, in Fig. 2C, we observed that other STRIPEs appeared inconsistently between the same samples mapped to the human genome or the parasite genome. This could potentially reflect some difference in how the reads are mapped to the genomes, or another difference. Consistent with that observation, we have found that the appearance and severity of the STRIPE can vary substantially with the methods used for alignment and quantification (Supplementary Fig. S5). Although we have not established a reproducible connection, batch effects or barcodes used in the construction of sequencing libraries could conceivably play a role (Supplementary Table S3). Without understanding the technical origin, it remains impossible to identify STRIPEs in single point or widely spaced temporal samples, which can easily introduce errors in the interpretation of these sparse transcriptomic data.

## Supplementary Material

gkaf524_Supplemental_Files

## Data Availability

The data underlying this article are available in the NCBI Gene Expression Omnibus (GEO; http://www.ncbi.nlm.nih.gov/geo/) with the following accession numbers: GSE270331 for the Yeast Cell Cycle 2 dataset and GSE270332 for the Human Cell Cycle dataset. Previously published data were used for this work and are available in the NCBI Gene Expression Omnibus (GEO; http://www.ncbi.nlm.nih.gov/geo/) with the following accession numbers: GSE80474 for the Yeast Cell Cycle 1 dataset [17], GSE8799 for the Yeast Cell Cycle 1 microarray datasets [9], GSE209877 for the Human–*Plasmodium* Developmental Cycle dataset [18], and GSE57683 for the STRIPE containing dataset from Wang *et al.* [19].

## Supplementary data

Supplementary data is available at NAR online.
