# Supplementary material for: Identification and correction of time-series transcriptomic anomalies
Source: Nucleic Acids Res. 2025 Jun 30;53(12):gkaf524. doi: 10.1093/nar/gkaf524 (PMC12207401; doi:10.1093/nar/gkaf524)
Supplement: gkaf524_Supplemental_Files [file gkaf524_supplemental_files.zip › Campione_NAR_2024_Supplement_reviewed_round2.docx]

# SUPPLEMENTAL MATERIAL


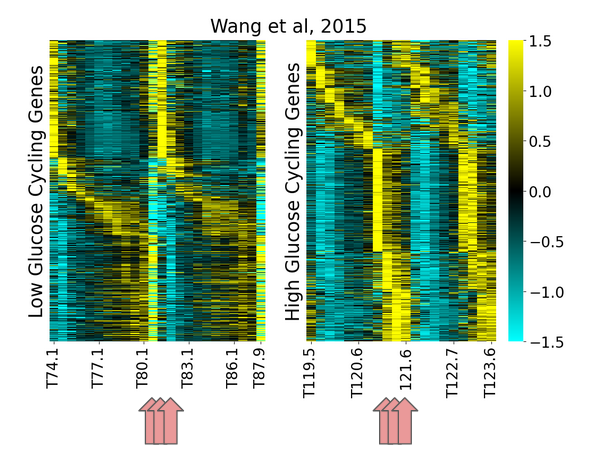


**Supplemental Figure 1: STRIPEs Identified in Wang et al., 2015.** STRIPEs were additionally identified in literature datasets outside of our lab in Wang et al. (Wang et al., 2015). STRIPEs identified by the detector function were highlighted with red arrows.


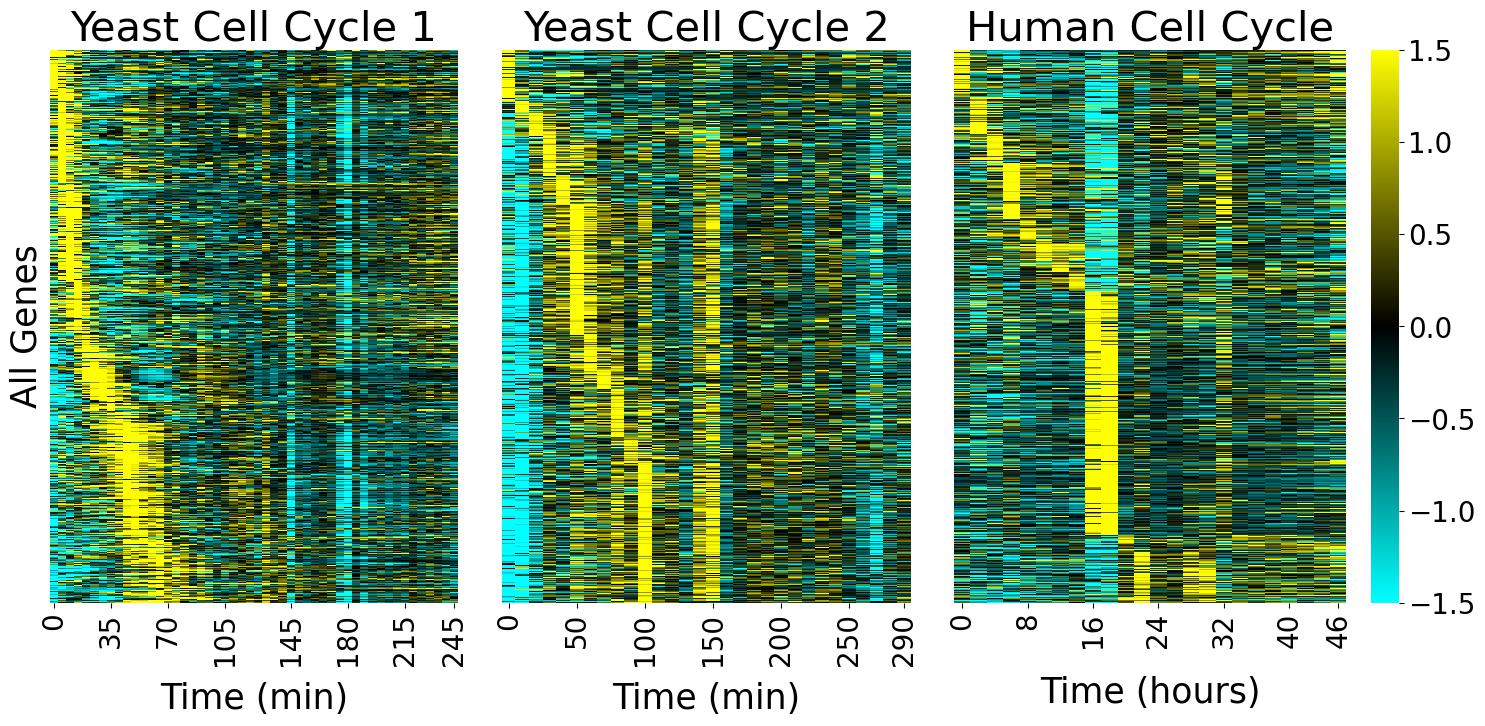


**Supplemental Figure 2: STRIPE Identification across the entire Transcriptome.** The entire transcriptome was plotted for each dataset in Figure 1a.


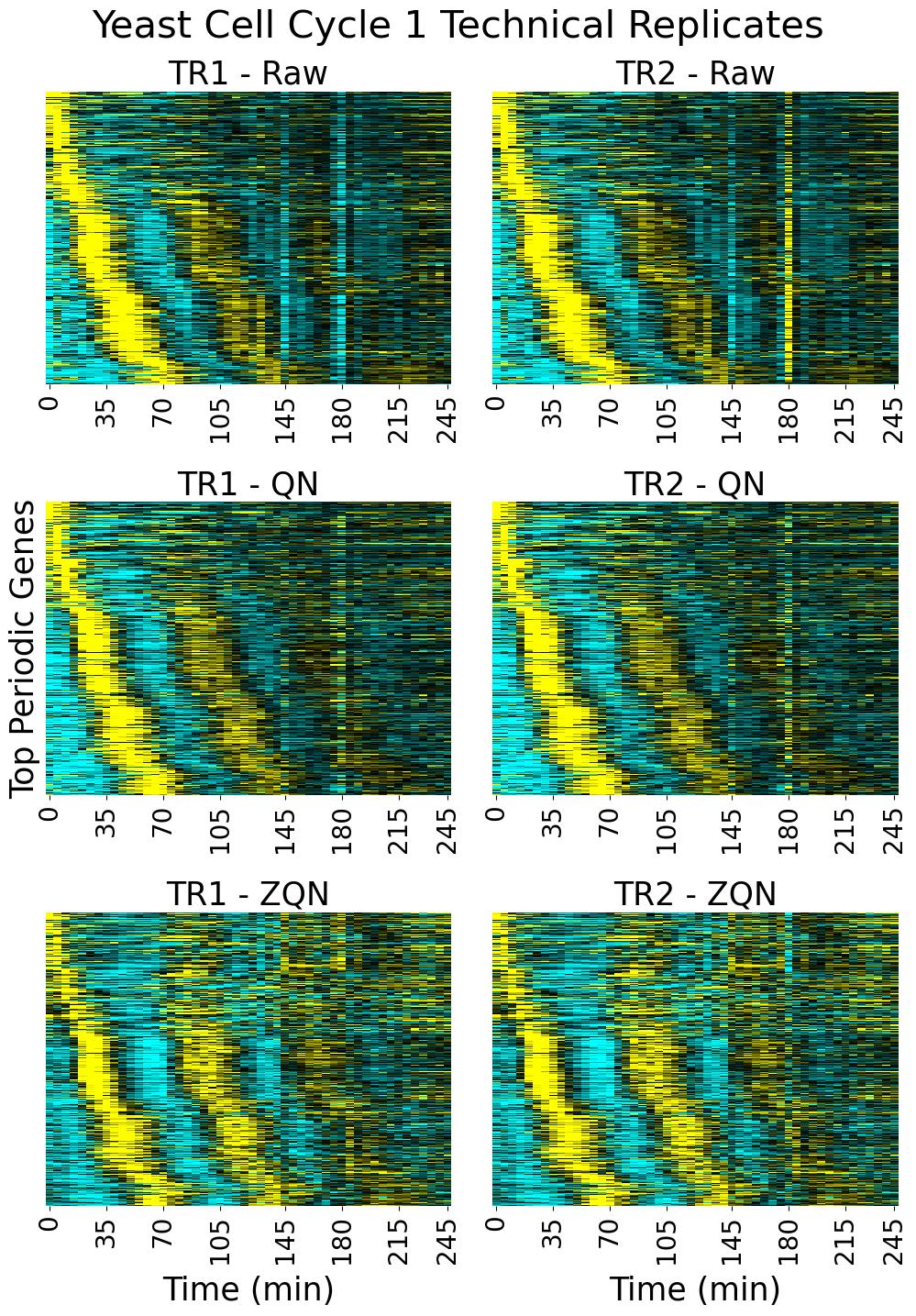


**Supplemental Figure 3: Technical Replicates Yeast Cell Cycle 1 Time-Series Show Different Expression Before STRIPE Correction.** Technical replicates 180 TR1 and 180 TR2 in the context of the entire Yeast Cell Cycle 1 time-series experiment. STRIPEs are corrected using quantile normalization (second row) and Z-score quantile normalization (bottom row).


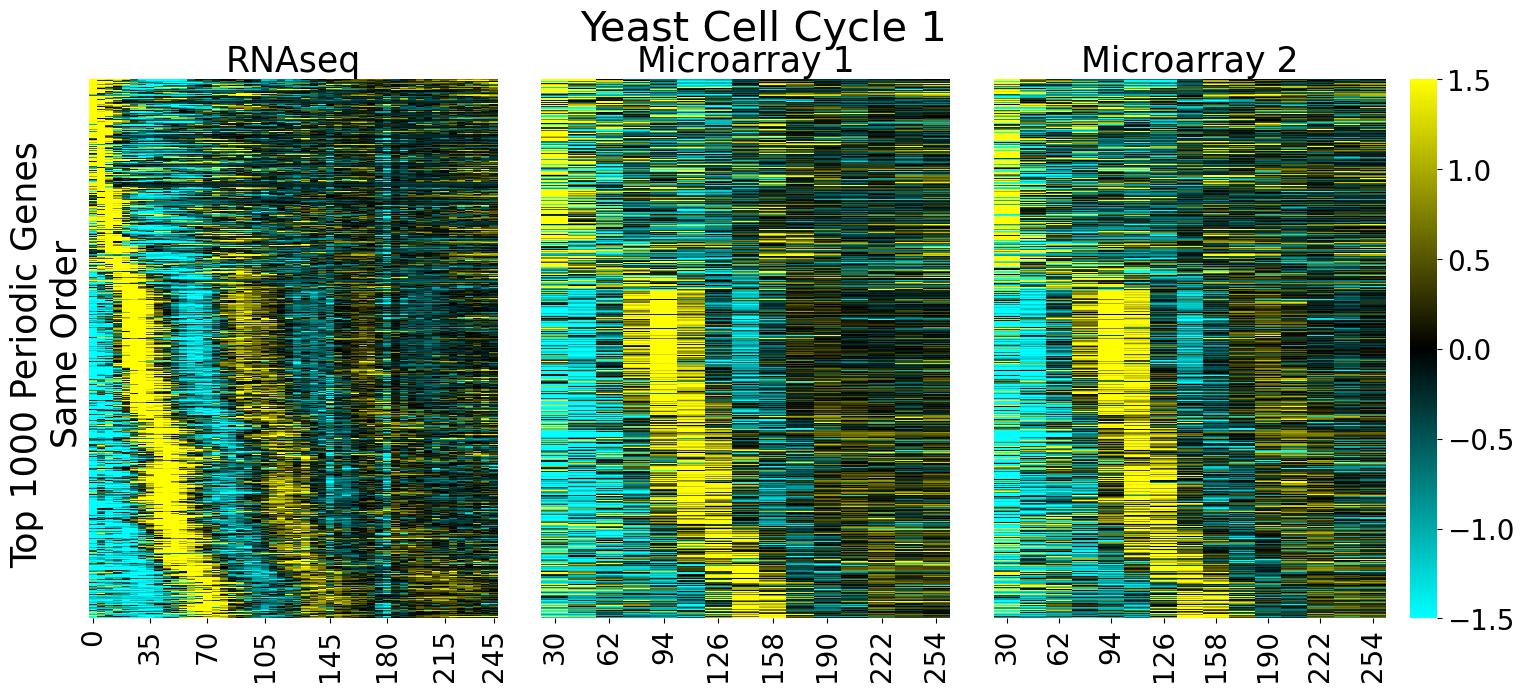


**Supplemental Figure 4: STRIPEs Present Only in One Experiment Across Replicates.** Yeast Cell Cycle 1 replicates across sequencing platforms (RNA-seq, Microarray 1, and Microarray 2). All three time-series experiments were performed in the same manner in optimal growth conditions and then processed using either RNA-sequencing or microarray. The same genes were shown in the same order for all three datasets. A STRIPE in the middle of the second cycle occurs only in one dataset, indicating it is not a biological transcriptional change.


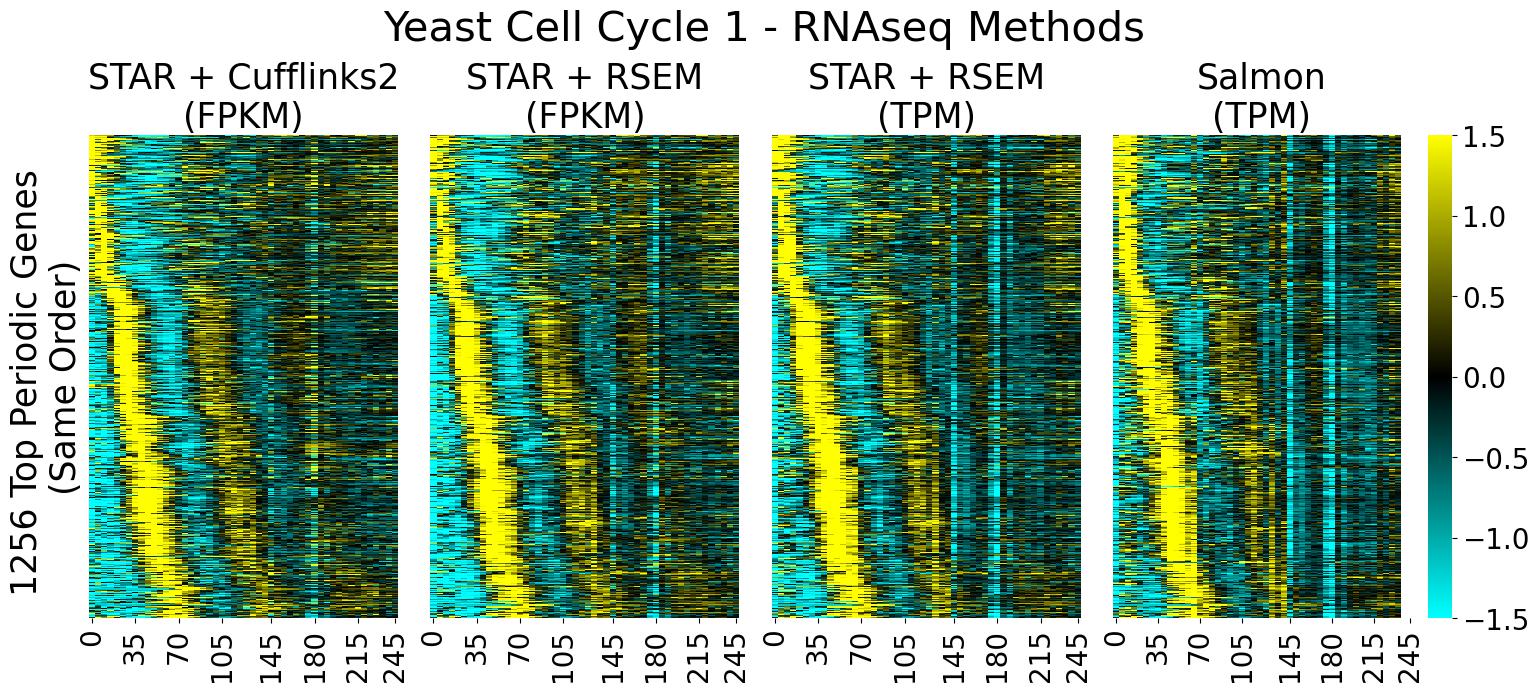


**Supplemental Figure 5: STRIPE Appearance Varies Across Different RNA-Sequencing Tools.** The RNA-sequencing raw data from the Yeast Cell Cycle 1 dataset was aligned and quantified using 3 different pipelines: STAR + Cufflinks 2, STAR + RSEM, and Salmon. As STAR + RSEM can produce both TPM and FPKM, both were compared. In each case STRIPEs are identified at timepoints 145, 175, and 180, however the severity in appearance varies across the different alignment and quantification pipelines. The STRIPEs present as bimodal STRIPEs only following alignment and quantification with STAR + Cufflinks2. Following alignment and quantification with STAR + RSEM for both FPKM and TPM, as well as for Salmon, produces lowered expression STRIPEs. Additionally, following alignment and quantification with Salmon, additional STRIPEs appear compared to the other RNA-sequencing pipelines.


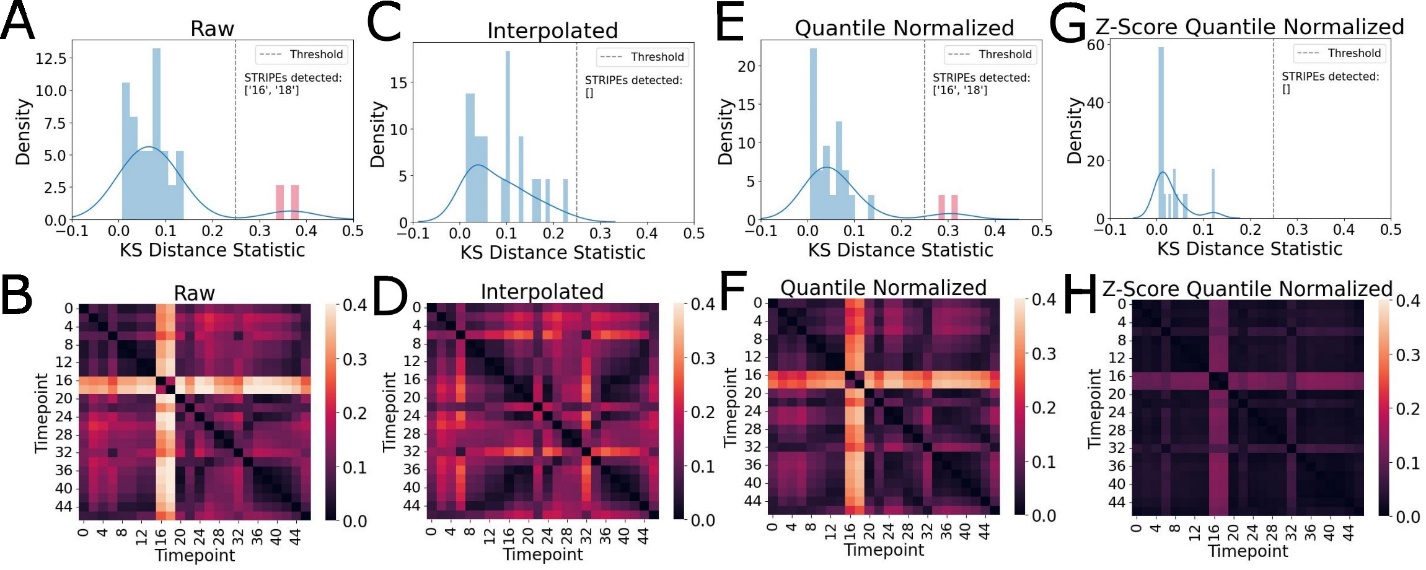

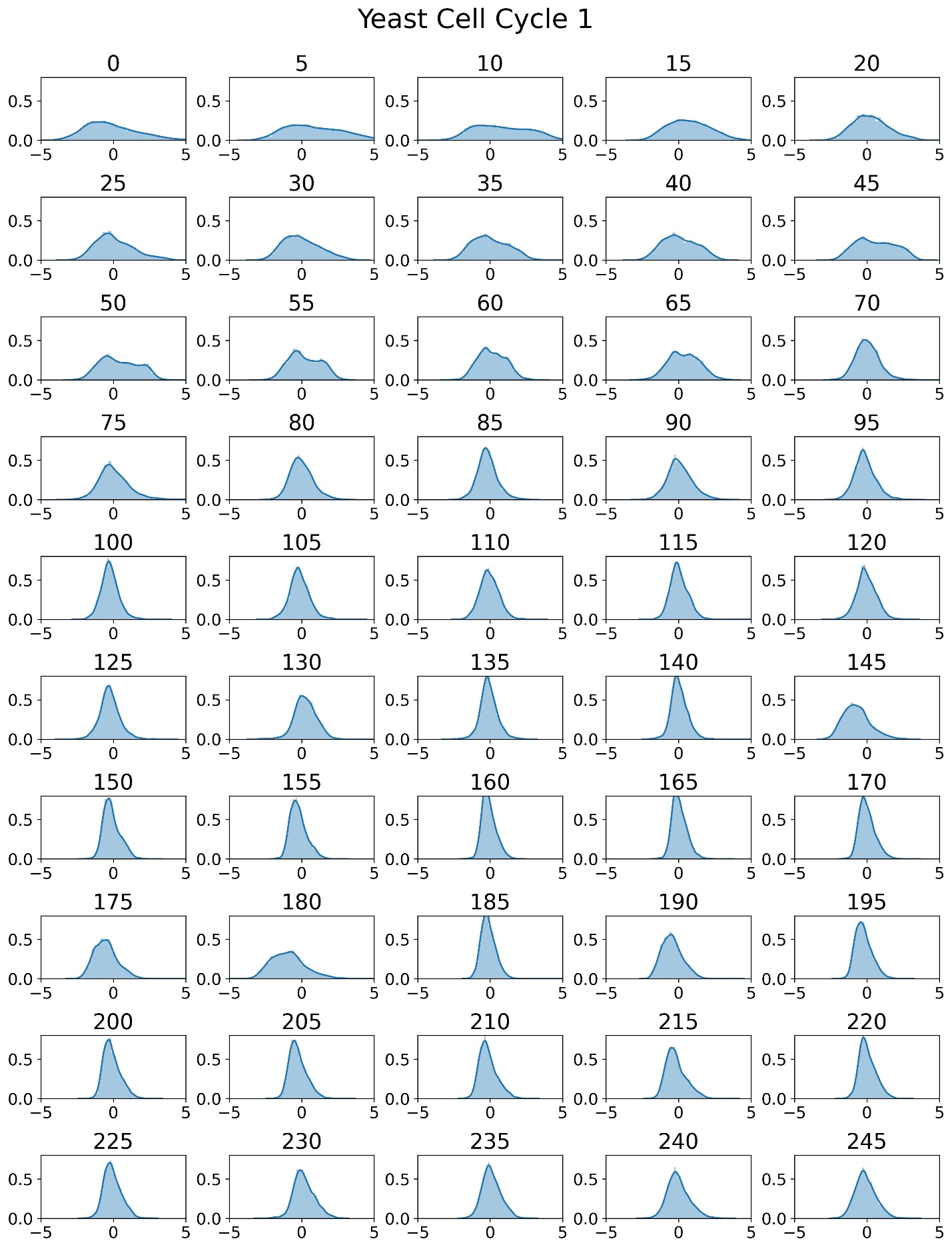

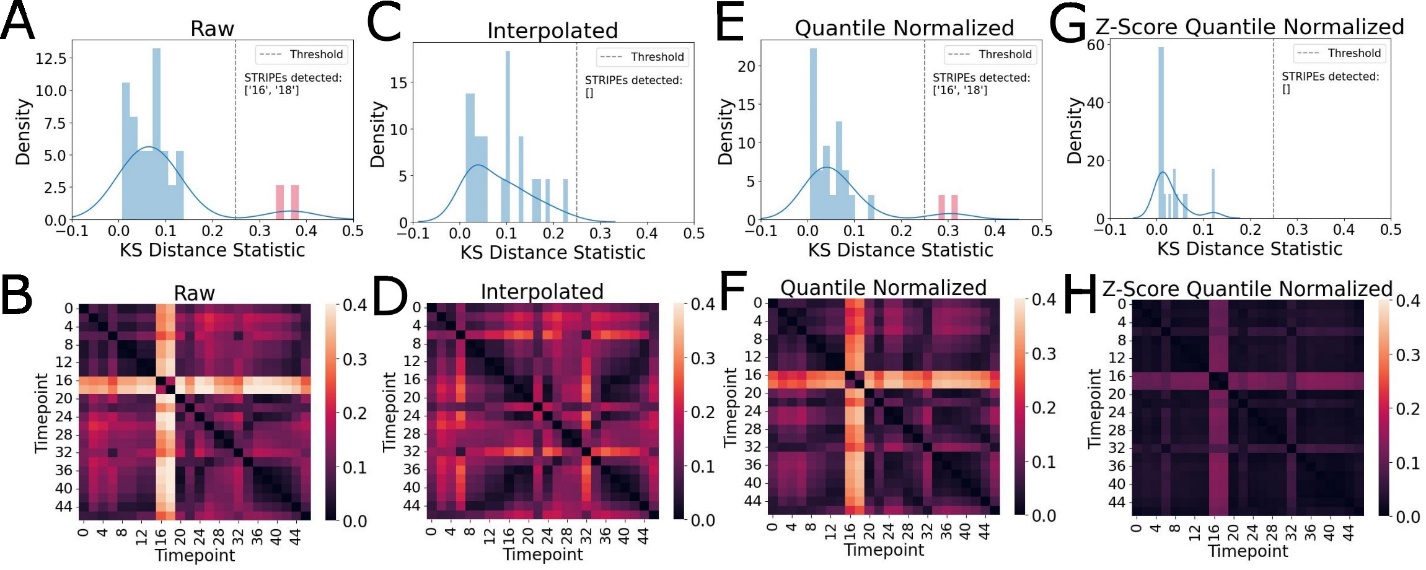

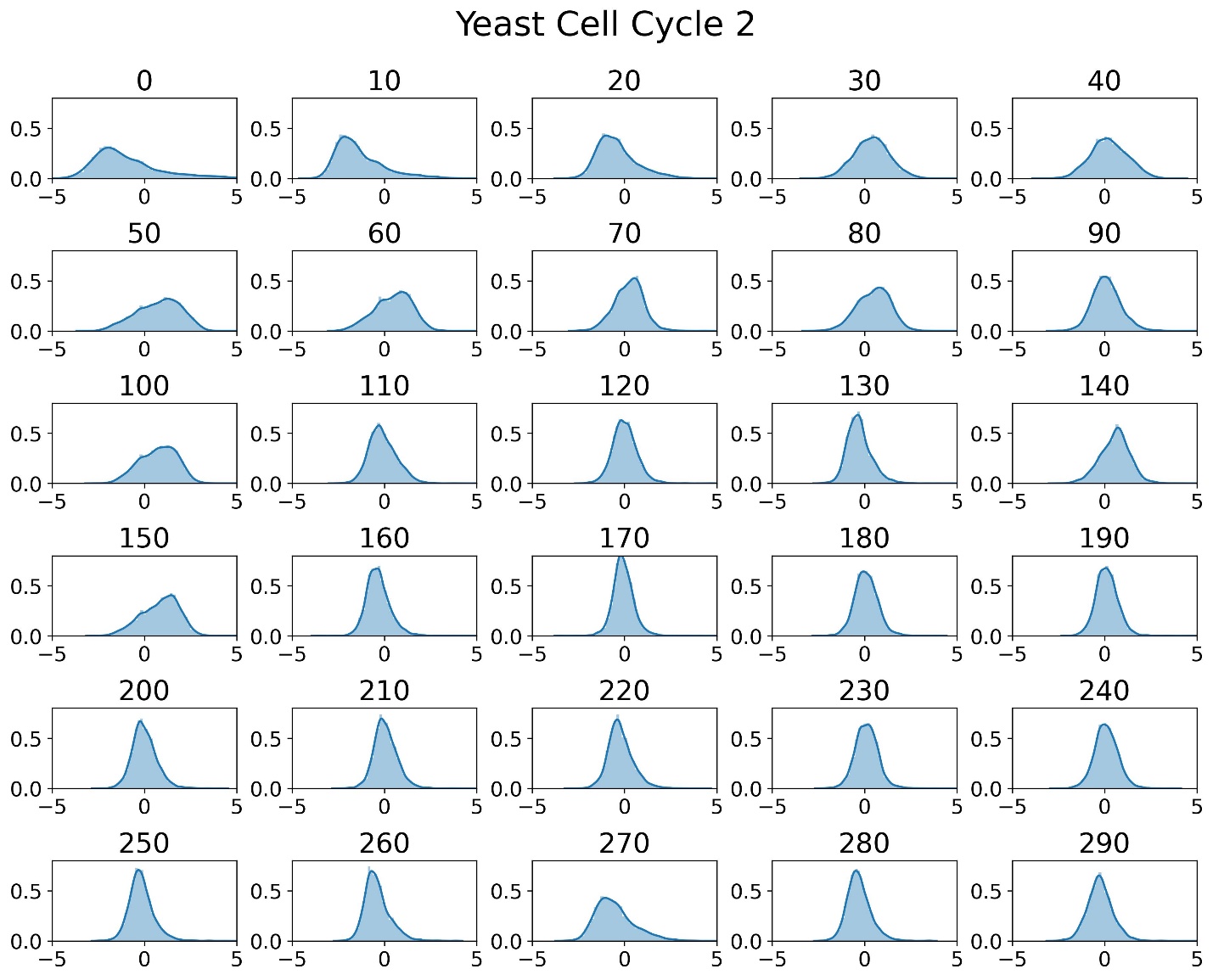

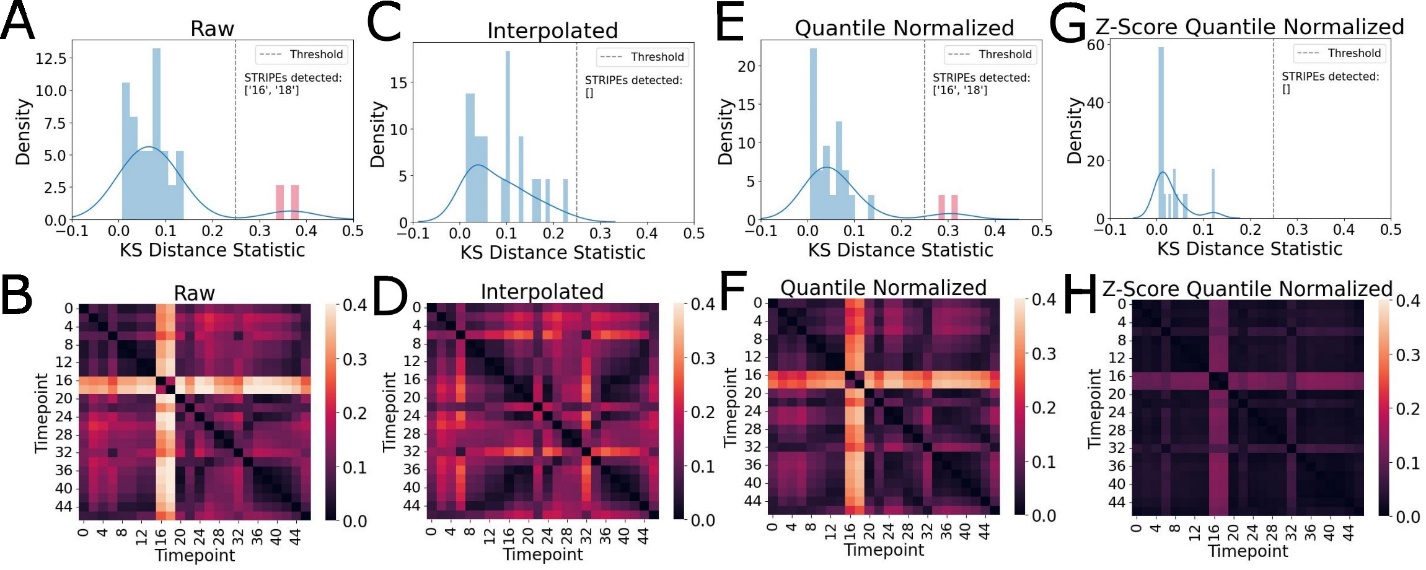

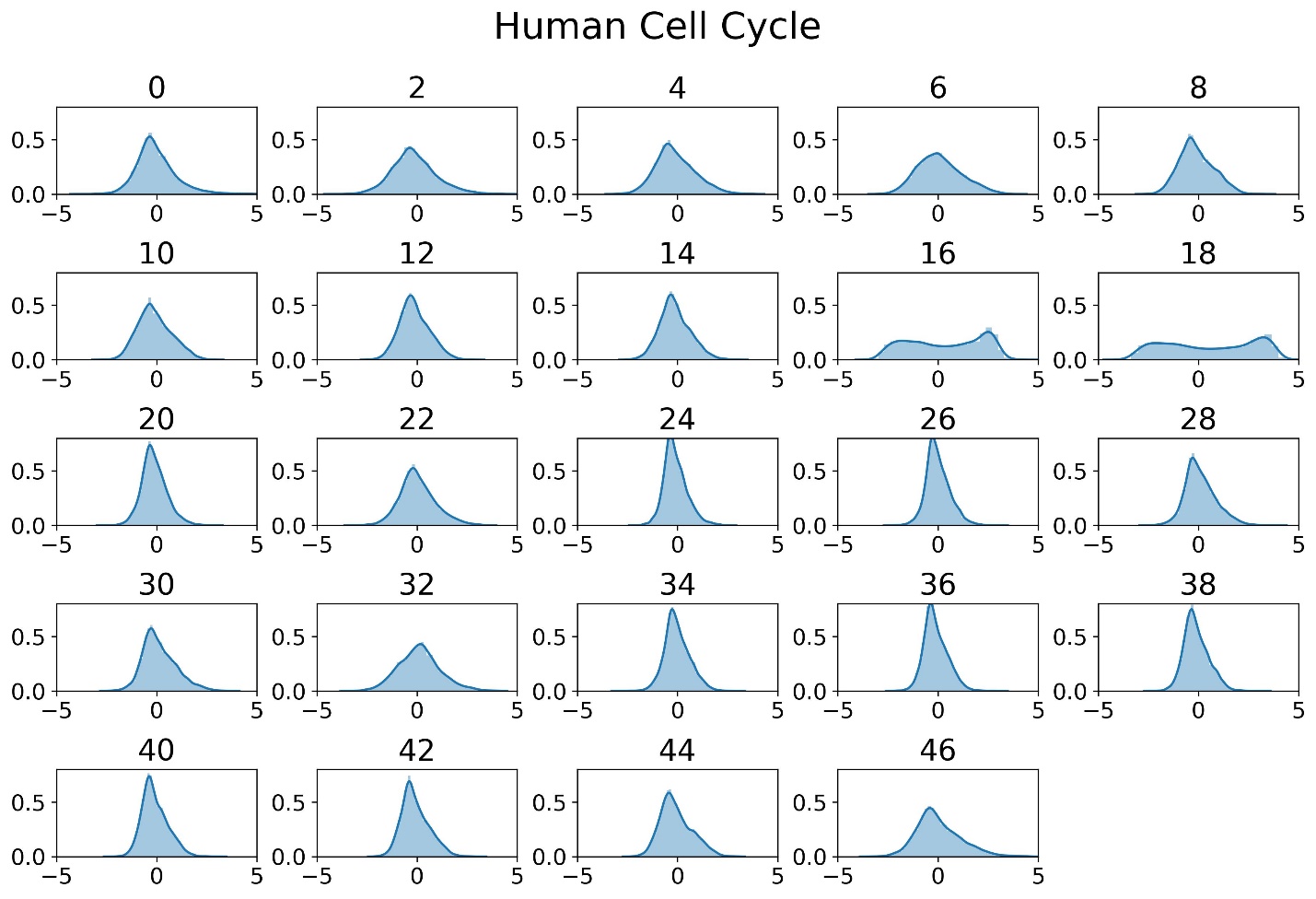
**Supplemental Figure 6: Z-score Expression Density Distributions.** Z-score normalized transcript level density distributions for every time point in A) the Yeast Cell Cycle 1 dataset, B) the Yeast Cell Cycle 2 dataset, and C) the Human Cell Cycle dataset.

**
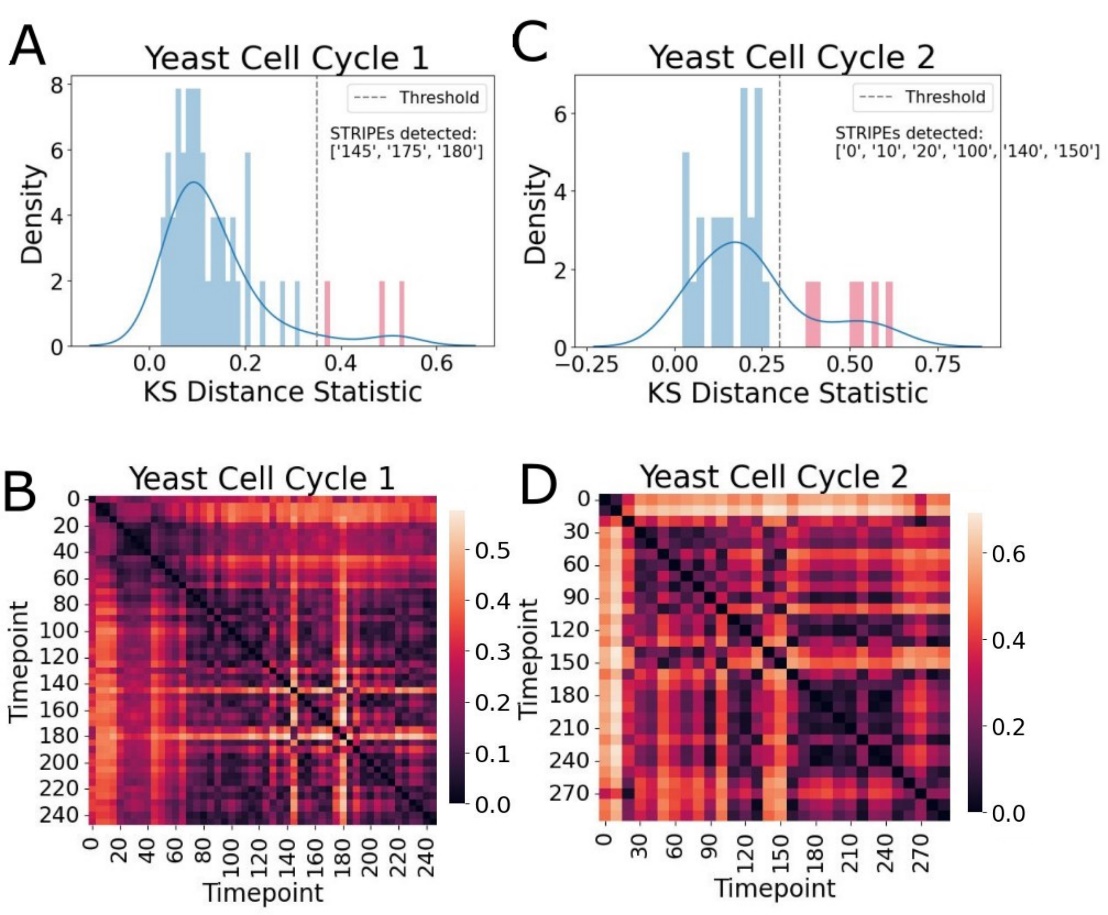
**

**Supplemental Figure 7: Kolmogorov-Smirnov Distance Comparisons for the Yeast Cell Cycle Datasets.** The distribution of KS distances for each comparison in the STRIPE detector (top row) and KS distance matrix heatmaps for each pairwise comparison (bottom row) for the Yeast Cell Cycle 1 (left column) and Yeast Cell Cycle 2 (right column) datasets. The KS distance threshold (dashed gray line) was selected by identifying outliers in the distribution.


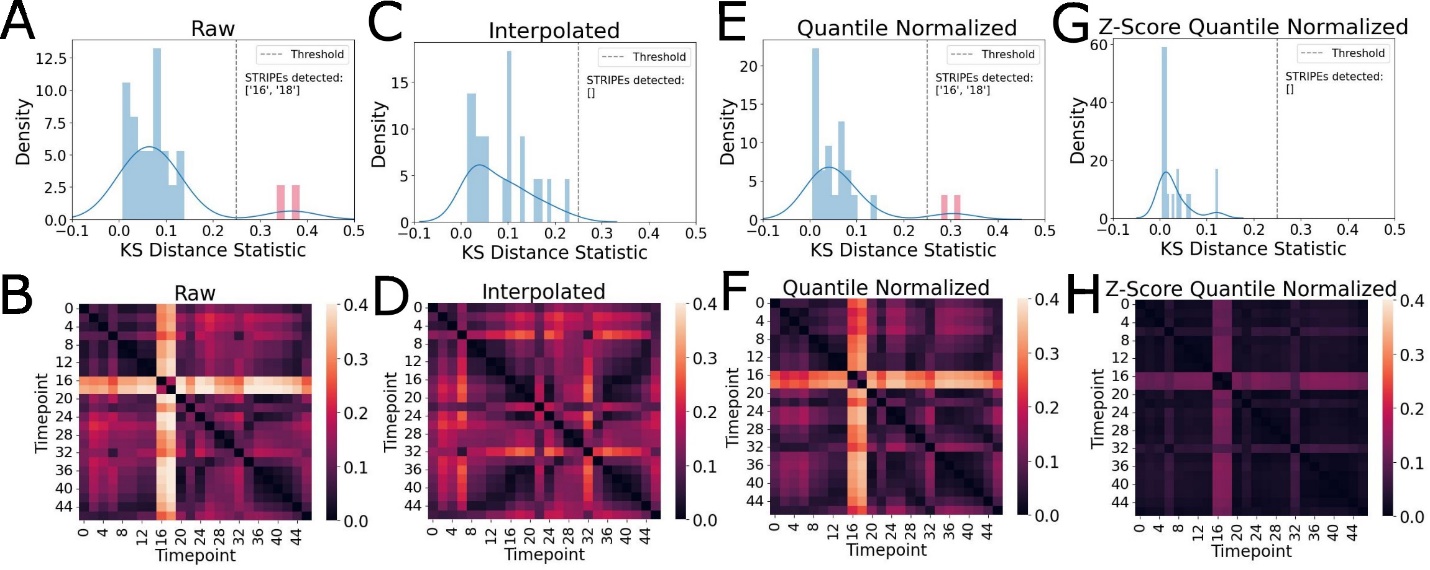


**Supplemental Figure 8: Kolmogorov-Smirnov Distance Comparisons for the Human Cell Cycle Dataset Following STRIPE Correction Methods.** The distribution of KS distances for each comparison in the STRIPE detector (top row) and KS distance matrix heatmaps for each pairwise comparison (bottom row) for the Human Cell Cycle raw data (first column), interpolated data (second column), quantile normalized data (third column), and z-score quantile normalized data (final column). STRIPEs were not detected after successful STRIPE correction via interpolation and z-score quantile normalization.


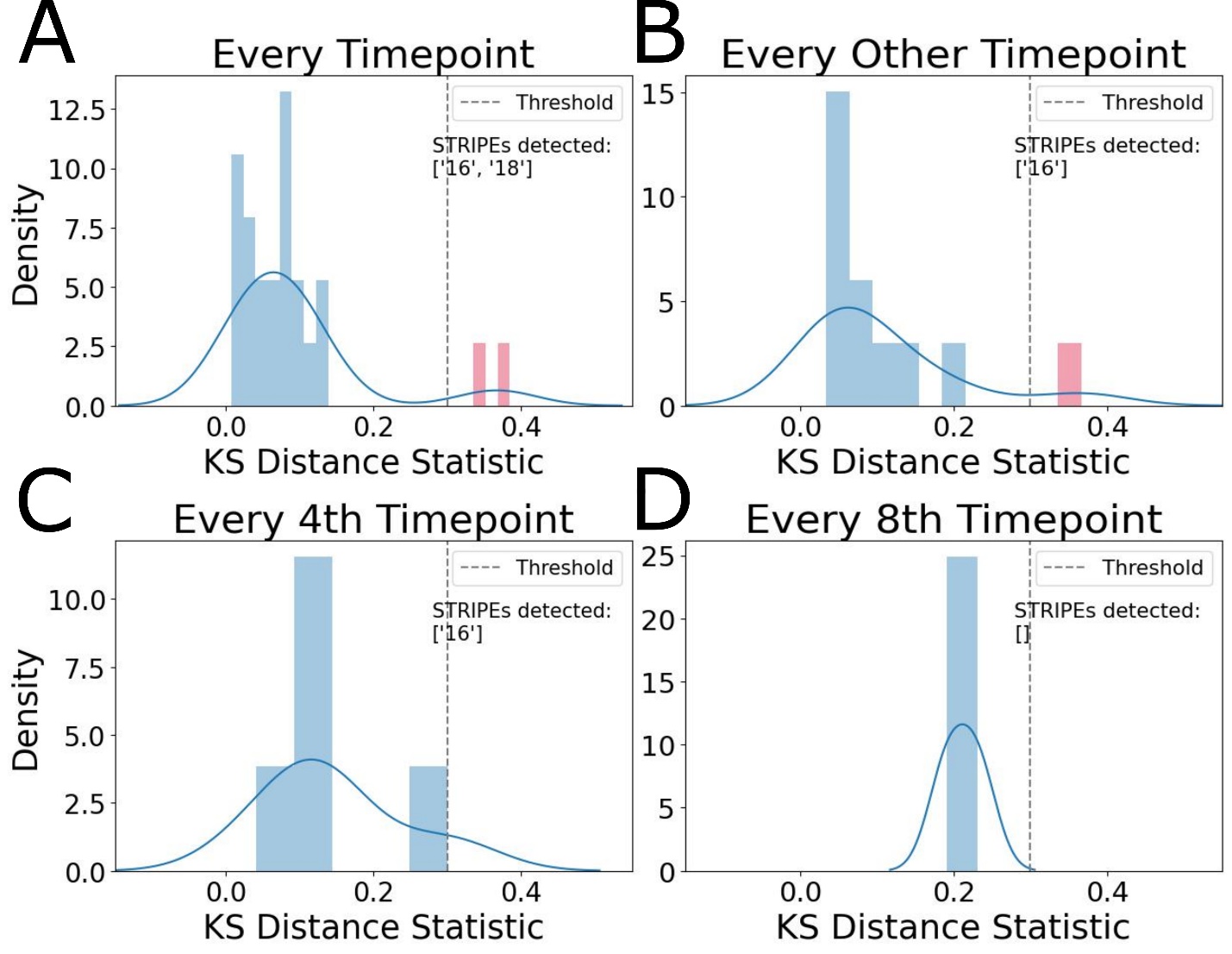


**Supplemental Figure 9: Sensitivity Testing for Kolmogorov-Smirnov Distance Comparisons for the Human Cell Cycle Dataset.** The distribution of KS distances for each comparison in the STRIPE detector for the Human Cell Cycle raw data with varying number of STRIPEs to show STRIPE detector performance on datasets with lower sample numbers. The KS distance distribution is shown A) for each timepoint in the HCC dataset [0, 2, 4, 6, 8, 10, 12, 14, 16*, 18*, 20, 22, 24, 26, 28, 30, 32, 34, 36, 38, 40, 42, 44, 46], B) for every other timepoint in the HCC dataset [0, 4, 8, 12, 16*, 20, 24, 28, 32, 36, 40, 44], C) For every fourth timepoint in the HCC dataset [0, 8, 16*, 24, 32, 40], and D) for every 8^th^ timepoint in the HCC dataset [0, 16*]. At least one STRIPE is present in each case (indicated with a *).

| **Dataset** | **Yeast Cell Cycle 1** | **Yeast Cell Cycle 2** | **Human Cell Cycle** | **Human-*Plasmodium* Developmental Cycle** | **Yeast Metabolic Cycle** |
| --- | --- | --- | --- | --- | --- |
| **Conditions** | YEP 2% Dextrose  30°C | YEP 2% Dextrose  38.5°C | RPMI + 10% BCS (bovine calf serum) + P/S | Whole ex vivo blood culture | High vs Low Glucose in Chemostat |
| **Cell Details** | *S. cerevisiae*  BF264-15D MATa bar1 | *S. cerevisiae*  BF264-15D MATa bar1 | K562 Cell Line | Sample 17 ex-vivo cultures of human blood infected with P. vivax | *S. cerevisiae* diploid CEN.PK |
| **Synchronization** | Alpha Factor | Alpha Factor | Centrifugal elutriation | NA | Chemostat culture |
| **Time Points** | 0-245 min  Every 5 min | 0 - 290, min Every 10 min | 0 - 46 hours,  Every 2 hours | 0 - 45 hours, Every 3 hours | T74.1 - T87.9 and T119.5 - T123.6 |
| **RNAseq Alignment/Quantification** | STAR + RSEM  (Additionally, STAR + Cufflinks & Salmon for comparison) | STAR + RSEM | STAR + Cufflinks/Cuffnorm | STAR + RSEM | NA |
| **Citation** | Kelliher et al., 2016 (RNAseq) Orlando et al. 2008 (microarray) | NA | NA | Motta et al., 2023 | Wang et al., 2015 |

**Supplemental Table 1: Description of Datasets.**

| **Comparison** | **Datasets** | **P-val** |
| --- | --- | --- |
| Total Reads | Yeast Cell Cycle 1 | 0.306 |
| Total Reads Aligned Uniquely to the Yeast Genome | Yeast Cell Cycle 1 | 0.381 |
| Total Reads Mapped to Annotated Genes | Yeast Cell Cycle 1 | 0.236 |
| RIN Score | Yeast Cell Cycle 1 | 0.0794 |
| RIN Score | Yeast Cell Cycle 2 | 0.0011 |
| RIN Score | Yeast Cell Cycle 1, 2 | 0.1033 |
| Concentration (ng/μl) | Yeast Cell Cycle 1, 2 | 0.6184 |
| 260/280 Ratio | Yeast Cell Cycle 1, 2 | 0.9911 |
| 260/230 Ratio | Yeast Cell Cycle 1, 2 | 0.8402 |
| PF* Yield (bp) | Yeast Cell Cycle 1, 2 | 0.7007 |
| Number of PF* Clusters** | Yeast Cell Cycle 1, 2 | 0.2644 |
| Q30% | Yeast Cell Cycle 1, 2 | 0.9695 |
| Average Quality Score | Yeast Cell Cycle 1, 2 | 0.9589 |
| total_reads | Yeast Cell Cycle 2 | 0.0194 |
| uniquely_mapped_percent | Yeast Cell Cycle 2 | 0.0647 |
| num_splices | Yeast Cell Cycle 2 | 0.0232 |
| mismatch_rate | Yeast Cell Cycle 2 | 0.8592 |
| multimapped_percent | Yeast Cell Cycle 2 | 0.7718 |
| multimapped_toomany_percent | Yeast Cell Cycle 2 | 0.3339 |
| unmapped_tooshort_percent | Yeast Cell Cycle 2 | 0.0226 |
| RSEM_Unalignable | Yeast Cell Cycle 2 | 0.2781 |
| RSEM_Alignable | Yeast Cell Cycle 2 | 0.0704 |
| RSEM_Total | Yeast Cell Cycle 2 | 0.1314 |
| RSEM_alignable_percent | Yeast Cell Cycle 2 | 0.0388 |
| RSEM_Unique | Yeast Cell Cycle 2 | 0.0635 |
| RSEM_Multi | Yeast Cell Cycle 2 | 0.1677 |
| RSEM_Uncertain | Yeast Cell Cycle 2 | 0.1931 |

**Supplemental Table 2: Results from T-test Analysis.** Paired t-test p-values for potential well-correlated STRIPE indicators.

| **Barcode_Sequence_(5'_to_3')** | **Reverse_Complement_Sequence_(5'_to_3')** | **Total_Mappings_to_S288C_Genome** | **Time_Point_Samples_with_Barcode_(minutes)** |
| --- | --- | --- | --- |
| ATTCCT | AGGAAT | 9069 | 0 |
| ACTTGA | TCAAGT | 8732 | 75, 115, 145, 180 |
| ATGTCA | TGACAT | 7777 | 135, 215 |
| CTTGTA | TACAAG | 7526 | 90, 165, 200 |
| AGTCAA | TTGACT | 7331 | 170, 205 |
| GCCAAT | ATTGGC | 6883 | 65, 105, 135 |
| ACTGAT | ATCAGT | 6879 | 40 |
| TAGCTT | AAGCTA | 6602 | 85, 125, 155, 190, 245 |
| TGACCA | TGGTCA | 6270 | 15, 55, 95, 225 |
| AGTTCC | GGAACT | 5313 | 175, 210 |
| CAGATC | GATCTG | 4718 | 70, 110, 140, 235 |
| ACAGTG | CACTGT | 4717 | 20, 60, 100, 230 |
| GTTTCG | CGAAAC | 4320 | 25 |
| GATCAG | CTGATC | 4216 | 80, 120, 150, 185, 240 |
| CGATGT | ACATCG | 4061 | 5, 45 |
| ATCACG | CGTGAT | 3329 | 0 |
| GAGTGG | CCACTC | 3126 | 35 |
| TTAGGC | GCCTAA | 2738 | 10, 50, 90 |
| GGCTAC | GTAGCC | 2638 | 45, 130, 160, 195 |
| CCGTCC | GGACGG | 1621 | 220 |
| GTCCGC | GCGGAC | 1331 | 180 |
| CGTACG | CGTACG | 841 | 30 |

**Supplemental Table 3 :  Illumina Barcodes Associated with STRIPE Samples.** Illumina barcodes and their reverse complement sequences map to the yeast genome with varying frequencies, and one particular barcode is associated with 2 STRIPE samples.

| **P-value Threshold** | **Number of Differentially Expressed Genes** |
| --- | --- |
| 0.05 | 2127 |
| 0.1 | 2674 |
| 0.2 | 3348 |

**Supplemental Table 4: DESeq2 Analysis on YCC1.** A python implementation of DESeq2 (Muzellec, 2023) was used to identify differentially expressed genes between STRIPE and non-STRIPE samples in the YCC1 dataset at three different p-value (padj) thresholds (0.05, 0.1, and 0.2).

# **ADDITIONAL SUPPLEMENTAL FILES:**

**Supplemental File 1:** Python utilities file containing functions for STRIPE detection and STRIPE correction.

For more information: <https://gitlab.com/haase-lab-group/stripe_anomalies>.

**SUPPLEMENTAL MATERIALS AND METHODS**

Quantile normalization was performed using a basic quantile normalization function on a pandas dataframe in Python. The function is shown below:


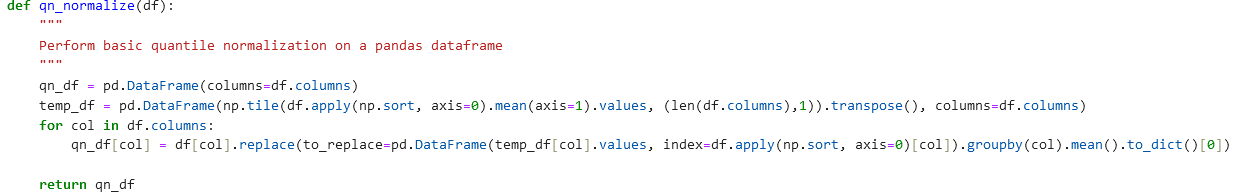


For Z-score quantile normalization the pandas dataframe is Z-score normalized using the Python scipy package prior to quantile normalization. Notably, the order of operations is important for successful STRIPE correction. When quantile normalization is applied followed by Z-score normalization (e.g. in Figure 4B QN where the data were first quantile normalized and then Z-score normalized for visualization) the STRIPEs can persist (as seen in the Human Cell Cycle dataset). However, when Z-score normalization is applied before quantile normalization (e.g. in Figure 4B ZQN where the data were Z-score normalized and then quantile normalized) the STRIPEs are successfully corrected in all datasets.

Order of operations is important in other biological measurements as well. For example, RPKM/FPKM and TPM differ in the order of operations (Olsen & Christensen, 2018). And this can have substantial effects. For example, in Supplemental Figure 5, using the same alignment and quantification tools, FPKM and TPM did show differences in STRIPE appearance.

The t-tests in Supplemental Table 2 were performed using a paired t-test in the R statistical programming environment (function t.test with argument: paired = T; R Core Development Team., 2017). They were applied to the Yeast Cell Cycle 1 and Yeast Cell Cycle 2 datasets independently, as these were collected using different methods, under different conditions, at different times. Therefore, each dataset had slightly different metrics that could be collected. For comparable metrics, t-tests were performed using samples from both datasets. All t-tests and their results can be found in Supplemental Table 2.

The DESeq2 analysis was implemented using a python implementation of DESeq2 (Muzellec, 2023). This analysis can be found on the Gitlab: <https://gitlab.com/haase-lab-group/stripe_anomalies>.
